# Bioinspired versus Enzymatic Oxidation of Some Homologous Thionine Dyes in the Presence of Immobilized Metalloporphyrin Catalysts and Ligninolytic Enzymes

**DOI:** 10.3390/ijms18122553

**Published:** 2017-11-28

**Authors:** Gianmarco Cocco, Andrea Cocco, Francesca Sollai, Enrico Sanjust, Paolo Zucca

**Affiliations:** Department of Biomedical Sciences, Biochemistry Unit, University of Cagliari, 09042 Monserrato (CA), Italy; gmarco.cocco@gmail.com (G.C.); andrewwww20@gmail.com (A.C.); sollai@unica.it (F.S.); pzucca@unica.it (P.Z.)

**Keywords:** thionine, biomimetic, metalloporphyrins, metalloporphines, peroxidase, peroxygenase, monopersulfate, hydrogen peroxide

## Abstract

Thionines are recalcitrant and polluting textile dyes presenting various degrees of *N*-methylation. In this paper, a complete series of homologous thionines was used as the substrates for oxidation in the presence of a bioinspired commercial iron-porphyrin immobilized on to imidazole- and pyridine-functionalized fumed silica, to emulate the active site of ligninolytic peroxidases. The obtained catalytic adducts showed a remarkable ability to catalyze thionine dye oxidation in the presence of different oxidants such as potassium monopersulfate and hydrogen peroxide. Different oxidation patterns were obtained and mechanistically discussed, in comparison with those observed in the presence of some ligninolytic oxidizing enzymes.

## 1. Introduction

**Figure 1:**
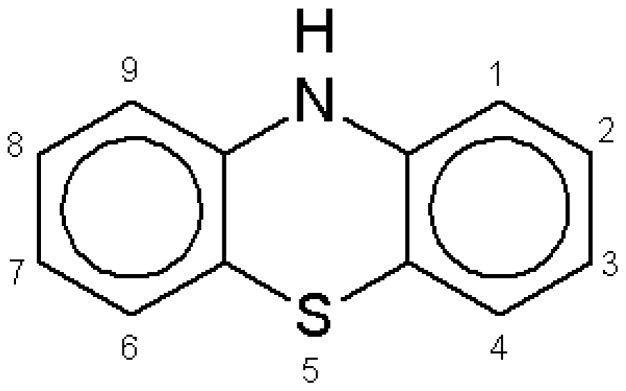
Fundamental structure of phenothiazines.

Within the vast family of thiazines, phenothiazines deserve attention because the fundamental molecule (Figure 1) and a huge number of its derivatives have a wide variety of applications, ranging from drugs to industrial dyes [1,2].

**Figure 2:**
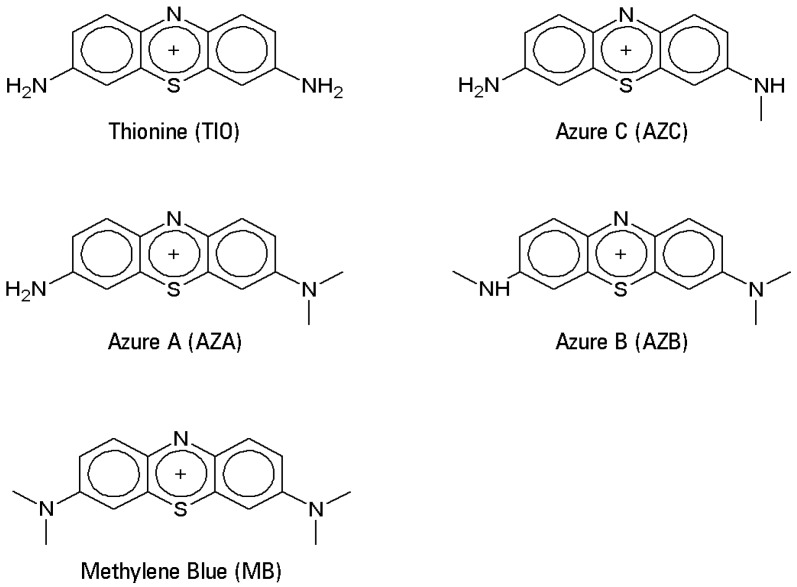
Azure C (monomethylated), Azure A (*N*,*N*-dimethylated), Azure B (trimethylated), and Methylene Blue (tetramethylated) are derivatives of thionine.

The phenothiazine family comprises—among many other substances—a number of water-soluble cationic blue dyes, sharing the 3,7-diamino-phenothiazinium structure, and largely used as industrial, biomedical, and histological dyes [3]. The mother compound is called thionine (also known as Lauth’s Violet, TIO), usually sold as the acetate salt. Within the family of the thionines, the two aminogroups can bear methyl substituents, giving rise to Azure C (monomethylated, AZC), Azure A (*N*,*N*-dimethylated, AZA), Azure B (trimethylated, AZB), and Methylene Blue (tetramethylated, MB) (Figure 2).

**Figure 3:**
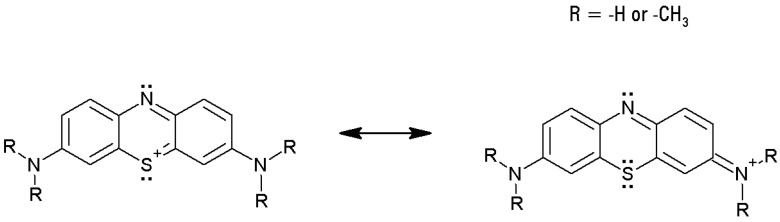
Electron resonance within a generic thionine.

The thionine dyes bear a net positive charge, delocalized along the sulfur atom and the two amine nitrogen atoms. Thus, on the whole, the thionines could be regarded as electron-poor tricycles. However, the heteroatoms (the sulfur, the nuclear nitrogen, two amines), formally bear unshared electron couples, and, therefore, those atoms could be considered as potentially electron-rich centers within a tricyclic cationic structure. Figure 3 shows some resonance forms for a generic thionine. This dual nature of the thionines has triggered the present study, which aimed to investigate the structure/reactivity relationship toward biomimetic oxidations mediated by a redox-active metalloporphyrin. In fact, the methyl groups show a mild electron-donating effect and, therefore, the basic character should increase from unsubstituted TIO to the fully methylated dye, MB.

The latter occupies a special place among the series, lacking any dissociable protons. As a consequence, it is sharply more basic than the other thionines and, moreover, it cannot be oxidized by any simple hydrogen atom transfer (HAT) mechanisms. In any case, regardless of the degree of methyl substitution, all thionines share a remarkable chemical stability, making their oxidation an interesting chemical challenge with potential applications [4,5]. In fact, oxidative and non-oxidative (hydrolytic) degradations of thionine dyes have drawn attention since a long time ago [6]. These otherwise stable compounds undergo chemical or enzymatic degradation under suitable experimental conditions. Progressive *N*-demethylation to unsubstituted TIO under alkaline conditions has been described [7], as well as both chemical [8] and enzymatic [9] oxidative degradations. In the present study, horseradish peroxidase, lignin peroxidase, manganese peroxidase, and fungal laccase were tested with respect to their catalytic efficiency in thionine dye degradation, and the obtained results were compared with those drawn from a series of experiments involving a ferriporphyrin-based heterogeneous bioinspired catalyst. The overall results were discussed with relation to the already published data and some mechanistic hypotheses were suggested. 

Porphyrins and their metal complexes (mainly with iron^II^ and iron^III^) are widespread in nature, and comprise various compounds differing in chemical character and specific position at the eight peripheral (β) positions of the macrocycle. Natural porphyrins invariably bear eight organic substituents at the β positions, whereas the four *meso* positions (the methyne carbons) all bear one hydrogen atom each. By contrast, the synthetic macrocycles, commonly obtained through the Rothemund reaction and its modifications and improvements (see [4] and citations therein), usually bear organic substituents just at the four *meso* positions, whereas the β positions bear hydrogen atoms or sometimes halogens, nitro groups, and so on, but not organic substituents. Following the directions of the International Union for Pure and Applied Chemistry (IUPAC) [10], such compounds should be named *porphines*, but the name porphyrins is so widespread that here the studied compounds are also referred to as porphyrins. Synthetic, unnatural metalloporphyrins—usually Fe^III^- or Mn^III^-porphyrins—have become very popular as biomimetic or at least bioinspired CYP450 emulators, even if only seldom are they able to use molecular oxygen [11] as the oxidizing agent to oxygenate their substrates. Very often, other suitable oxygen donors are effective, such as iodosobenzene, *t*-butyl-hydroperoxide, or hydrogen peroxide [4,12]. Therefore, in particular, when the oxidizing agents are hydrogen peroxide or other organic hydroperoxides, redox-active metalloporphyrins should be regarded as peroxygenase [13,14] or also peroxidase [15], rather than CYP450 emulators. The main features of redox-active metalloporphyrins as bio-inspired emulators of such enzymes have been recently reviewed in the view of technological applications [4,12], being able to oxidize several recalcitrant compounds of industrial interest, including non-phenolic aromatic compounds [12], halogenated phenolics [16], and textile dyes [17,18,19].

**Figure 4:**
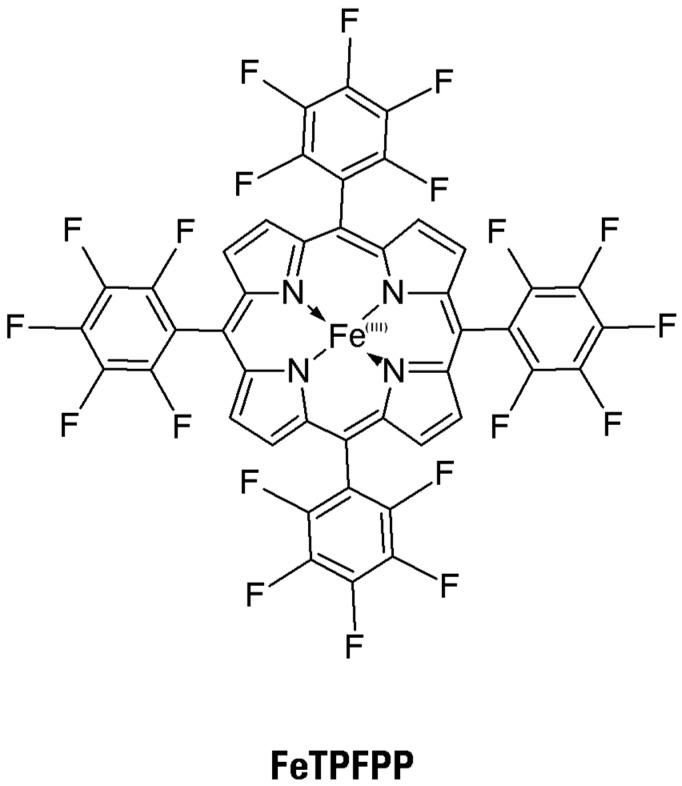
Metalloporphyrin 5,10,15,20-tetrakis-pentafluorophenyl-porphyrinato-iron^III^ (FeTPFPP) used in this study.

In the present study, a redox-active metalloporphyrin was used, namely 5,10,15,20-tetrakis-pentafluorophenyl-porphyrinato-iron^III^ (FeTPFPP) (Figure 4). This was immobilized via a coordination bond between the iron center and pyridyl (and also imidazolyl) moieties, covalently fixed onto a water-insoluble but hydrophilic support, to obtain heterogeneous catalysts which were tested toward the five thionine dyes, in an aqueous environment. The choice of coordination bond as the immobilization approach allows the mimicking of the redox active site of ligninolytic peroxidases, provided that this approach dramatically increases the catalytic performances of immobilized metalloporphyrins [12,20,21,22,23].

The support chosen for the immobilization was fumed silica (FSG). This is a particular form of amorphous silicon dioxide, obtained by burning at very high temperatures SiCl_4_ injected within a hydrogen flame [24]. Under such conditions, the formed silica liquid droplets suddenly solidify to generate almost spherical nanoparticles that, in turn, aggregate, forming superstructures. This is an amorphous, non-porous, hygroscopic, and quite hydrophilic material, with a very high surface/volume ratio and a high surface concentration of silanol groups, responsible for its strong adsorptive properties [25]. On the other hand, the high density of surface silanols allows facile surface modifications by a number of different chemical agents, such as organosilanes [26]. To our knowledge, this is the first report of both fumed silica use in metalloporphyrin immobilization procedures, and of a systematic study about the oxidation by metalloporphyrins of thionines with various degrees of methylation.

## 2. Results

Fumed silica has only seldom been used as a support to obtain heterogenized catalysts [27,28]. Commercial fumed silica is a free-flowing fine powder, apt for biomolecule immobilization after functionalization. For this purpose, a long-chain polyaminosilane was used to prepare a modified fumed silica that maintained its sharply hydrophilic character, whereas its original slightly anionic behavior was overwhelmed by the sharply cationic features arising from the amine functions. Functionalization proceeded with high yields, as shown by indirect measurements of total nitrogen: up to 240 μEq/g of –NH_2_ groups.

**Figure 5:**
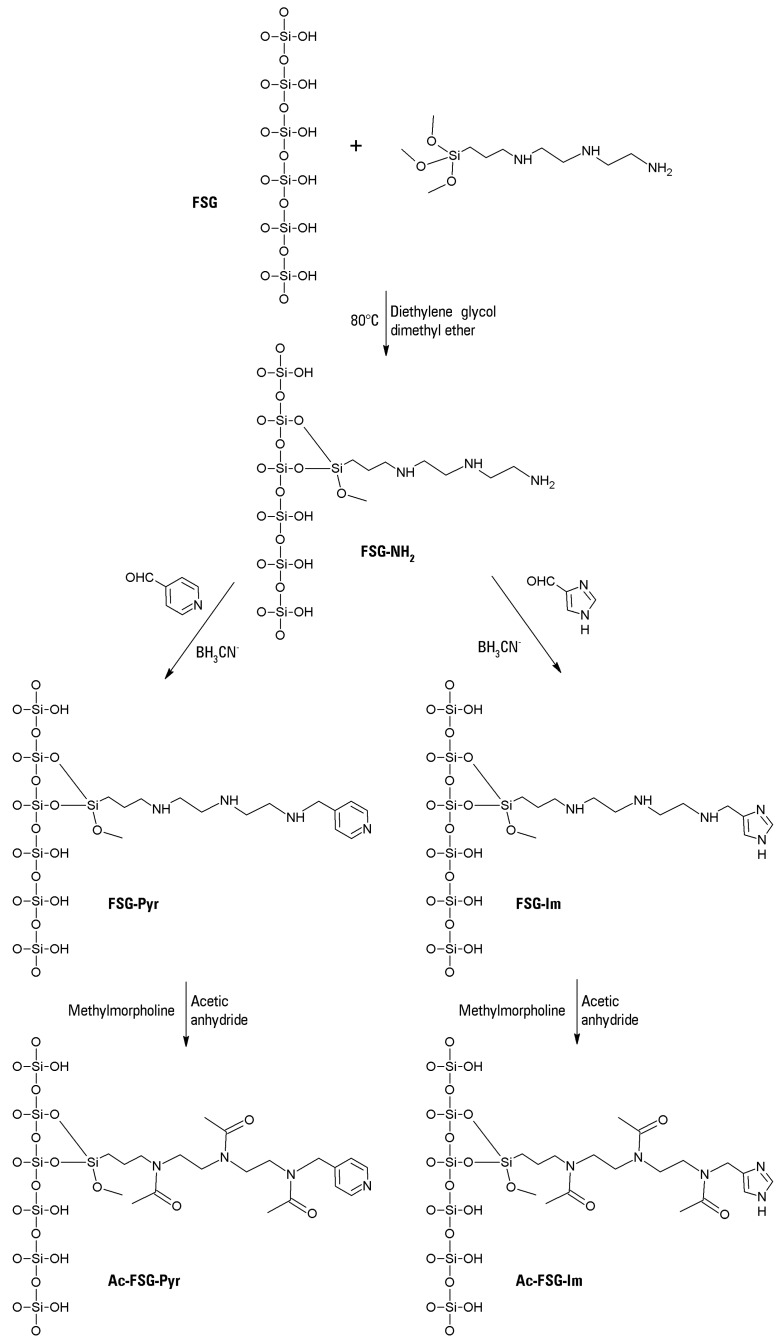
Putative structures of the four supports used in this study. Fumed Silica (FSG); Amine-grafted FSG (FSG-NH_2_); Pyridine-grafted fumed silica (FSG-Pyr); Imidazole-grafted fumed silica (FSG-Im); Acetylated FSG-Pyr (Ac-FSG-Pyr); Acetylated FSG-Im (Ac-FSG-Im).

In the subsequent step, these –NH_2_ groups nearly disappeared (reaction yield > 99%) upon reductive alkylation with pyridine-4-carboxaldehyde (or imidazole-4(5)-carboxaldehyde). The long space arms with their polycationic nature prevented any adsorption of the tested cationic dyes on to the fumed silica surface. The primary amine termini of the space arms readily reacted with aldehydes to form Schiff bases, which were in turn reduced to their stable secondary amine derivatives by NaBH_3_CN [29]. This ability was exploited to add a pyridine ring (or an imidazole ring) to the end of the space arm, therefore providing a binding function for one axial position of the Fe^III^ center within the porphyrin macrocycle. For certain experiments, however, the cationic character of the silane space arm was abolished by acetylation, to study possible differences in the bleaching reactions, due to support fouling. The pyridyl (or imidazyl) moiety was able to bind the chosen metalloporphyrin via a coordination bond, as it remained untouched upon acetylation. All the transformations of plain fumed silica to the four possible heterogenized catalysts are presented in Figure 5.

In a first series of preliminary catalytic experiments, both imidazole- and pyridine-grafted fumed silicas (FSG-Im and FSG-Pyr, respectively) were used to immobilize FeTFPP. The results showed that imidazole-bearing supports led to a less efficient catalyst, in accordance with what has already been described about similar heterogeneous catalysts [20]. For instance, FSG-Im/FeTPFPP gave only 19% bleaching of thionine in 10’, whereas in the same time FSG-Pyr/FeTPFPP gave 56%. This is probably due to the higher electron deficiency of pyridine, which causes increased *Compound I* (*Cpd I*) analogue reactivity. In accordance with these data, only pyridine-bearing supports were used in the rest of the study.

The shape and volume distribution as well as the surface chemical changes of the silica particles, along the modification reactions, were checked and confirmed by scanning electron microscopy (SEM) and Fourier Transform Infrared (FT-IR) spectrometry.

**Figure 6:**
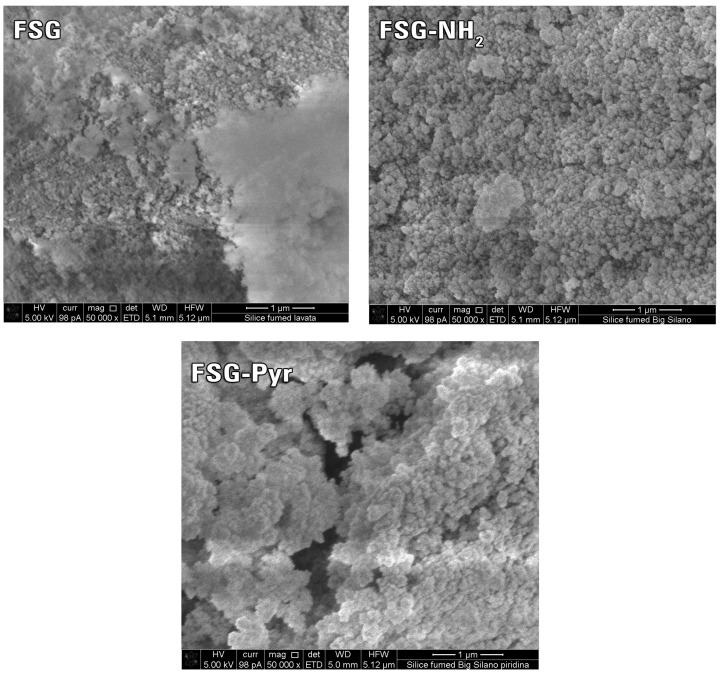
SEM images of FSG, FSG-NH2, and FSG-Pyr.

SEM images of FSG, FSG-NH_2_, and FSG-Pyr are shown in Figure 6, showing that only minimal changes occurred and that nanostructures were still within the nanoscale range (≤100 nm). Some aggregation of the particles seems to follow derivatization. Particularly, mean particle size increased from 61 nm (FSG) to 82 (FSG-NH_2_) and 123 nm (FSG-Pyr), but was compatible with metalloporphyrin immobilization.

FT-IR spectra showed small but definitive changes following the silanization, as already reported for similar supports [20] (Supplementary Figure S5). Particularly, new bands arose around 1550 cm**^−^**^1^ and 2900 cm**^−^**^1^ in FSG-Pyr, possibly deriving from heteroaromatic rings or –NH– bending [20].

**Figure 7:**
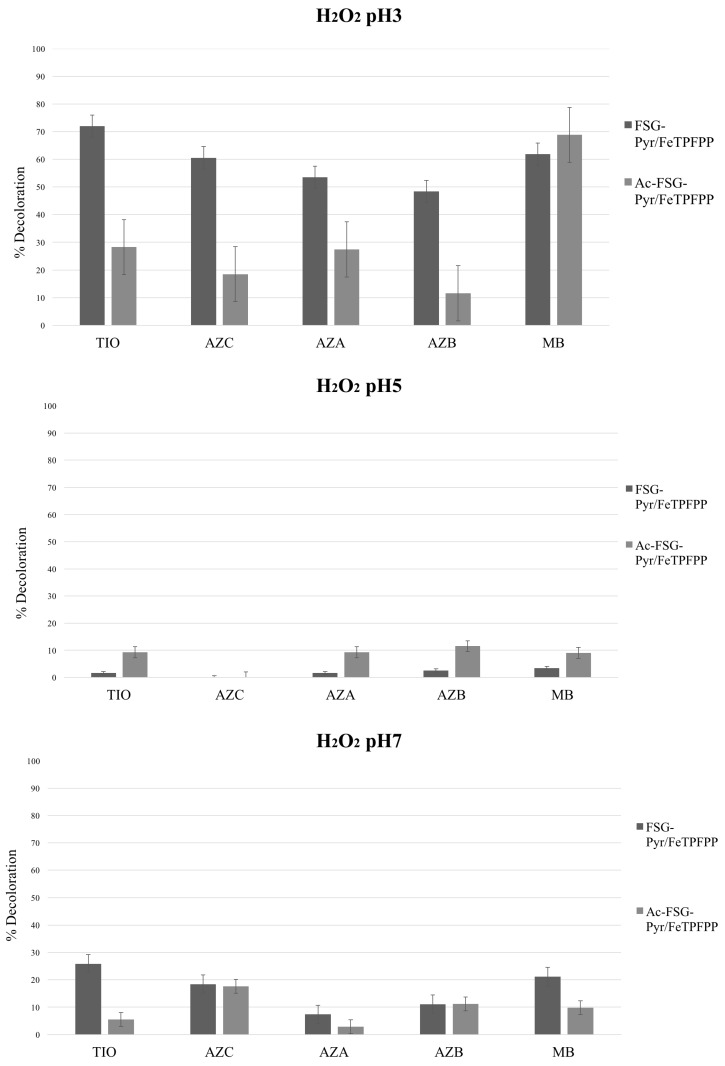
Catalytic activity of the tested immobilized metalloporphyrins in the presence of hydrogen peroxide as the oxidant at different pH values. Catalytic activity is expressed as % decolorization (i.e., % of initial absorbance at λ_max_ which disappeared during reaction). *n* = 3.

**Figure 8:**
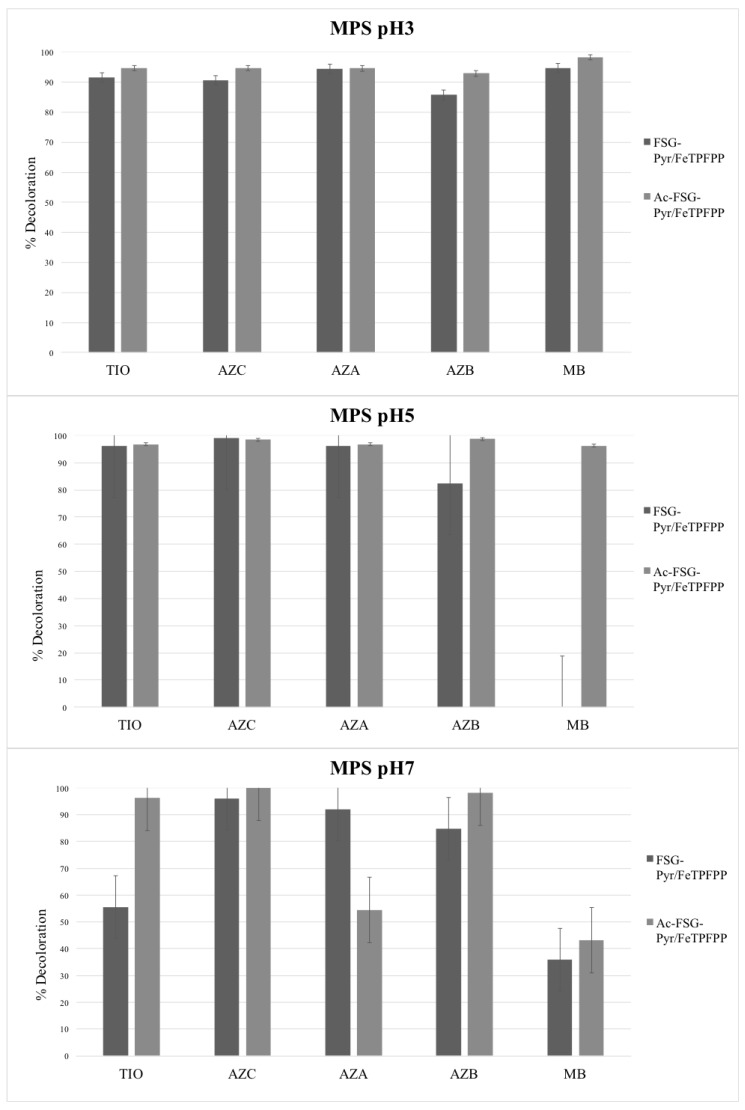
Catalytic activity of the tested immobilized metalloporphyrins in the presence of potassium monopersulfate (MPS) as the oxidant at different pH values. Catalytic activity is expressed as % decolorization (i.e., % of initial absorbance at λmax which disappeared during reaction). *n* = 3.

Bleaching experiments were carried out at different pH values and using two oxidizers, i.e., hydrogen peroxide and potassium monopersulfate (MPS). FeTPFPP was used (loading 6.7 μEq/g), immobilized on acetylated or non-acetylated pyridine-grafted supports. Thus, two catalysts were generated, differing by their neutral or cationic character. The results are presented in Figure 7 and Figure 8.

### 2.1. Hydrogen Peroxide

As a matter of fact, H_2_O_2_ in the absence of catalysts is a very poor oxidant toward the thionines; however, some studies have shown that under Fenton-like conditions, these dyes could be bleached [30,31,32].

The maximum activity was always observed at pH 3, whereas only a low level of activity could be found at pH 5 and pH 7. This was a general pattern, regardless of the particular catalyst and thionine dye. Such behavior strictly parallels that observed for Class II peroxidases [33]: hydrogen peroxide reacts with the Fe^III^ center within the porphyrin macrocycle leading to the *Compound zero* (*Cpd 0*), where a hydroperoxide anion HO–O**^−^** is bound to the metal ion [34]. At low pH values, the PorphFe^III^–O–OH group undergoes facile protonation and upon heterolytic scission, assisted by the imidazole moiety of the distal histidine, produces PorphFe^IV+^=O (*Cpd I*) and water. As a consequence, the optimal pH value for such peroxidases is—not surprisingly—around 3. In the case of iron-containing bioinspired metalloporphyrins, where no distal histidine is present, the same *Cpd 0* analogue can undergo an alternative fission (homolytic cleavage of the O–O bond, favored by low H^+^ concentrations), affording PorphFe^III^–O˙ (the radical form of the *Compound II* (*Cpd II)*, usually written as PorphFe^IV^=O) and hydroxyl radical HO˙ [35]. The latter is extraordinarily reactive and can attack the same ferriporphyrin, leading to catalyst bleaching and irreversible inactivation [36]. The *Cpd II* as an oxidizing agent is decidedly less active than *Cpd I* [37], which explains the quite poor activity observed at pH 5 and pH 7.

**Scheme 1:**
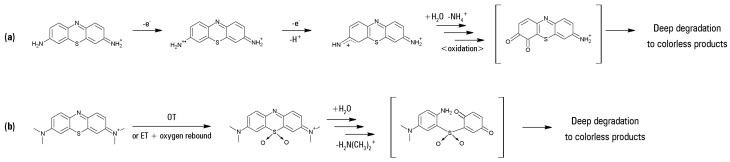
When hydrogen atoms are present, a hydrogen atom transfer (HAT) mechanism can be inferred (path **a**). On the contrary, the absence of a removable proton (as in the case of MB) suggests a direct attack to the sulfur atom through an electron transfer/oxygen transfer (ET/OT) mechanism (path **b**).

In detail, activity steadily decreased from TIO to AZB, therefore suggesting a HAT mechanism (as TIO bears four removable protons whereas AZB has only one). Upon hydrogen atom removal, an extremely reactive cationic radical should arise—as already suggested in the case of phenosafranine [18], which is quite prone to a further one-electron oxidation to a strongly electrophilic nitrenium cation. This, in turn, would undergo nucleophilic attack by water followed by subsequent hydrolysis and oxidation steps passing through an *ortho*-quinone and finally leading to rapid cleavage of the dye molecules (Scheme 1 path a).

MB lies quite out of this series, being—somewhat surprisingly—a good substrate: therefore, as it does not bear any removable proton, different mechanisms (Electron Transfer, ET or Oxygen Transfer, OT) must operate in this case. In particular, as already found in the case of sulfide ions [21] and thioethers [38,39,40,41], some redox-active metalloporphyrin oxo-derivatives are well capable of attacking the sulfur atoms, leading to sulfate or to sulfoxides/sulfones, respectively. The lack of any removable proton in the MB molecule could drive the catalyst action toward such a direct attack to the sulfur atom, therefore breaking the electron delocalization along the thionine nucleus with concomitant absorption loss in the visible region. This hypothesis is corroborated by the early observation by MacNeal et al. [6] who noted the facile oxidation of MB to the corresponding sulfone by an oxidant as feeble as FeCl_3_. Very recently, Mesquita et al. [31] have found a sulfoxide intermediate along the oxidative degradation of MB in heterogeneous Fenton-like conditions. Especially in the case of the arising sulfone, the strong electron-withdrawing effect should activate the immonium-bearing ring toward nucleophilic attack (by water) and subsequent fast oxidative/hydrolytic disintegration (Scheme 1, path b).

Moreover, an alternative hypothesis could afford a reasonable explanation for the almost complete bleaching of the studied dyes, without any evidence of stable colored intermediates: Oliveira et al. [32] have found evidence of hydroxylated intermediate formation along the bleaching process of MB under Fenton-like conditions. A progressive hydroxylation of the aromatic rings of the dye took place, producing more and more oxidizable intermediates which cannot accumulate and which explain the complete bleaching of the visible part of the optical absorption spectrum within two hours.

A similar bleaching pathway could well be operating under the reaction conditions described here, with the obvious difference that the oxidative attack on the aromatic rings of the dyes has to be electrophilic and not radical.

Acetylation of the polyamino silane bridge, by abolishing its positive charges, should facilitate the substrate approach and, therefore, should enhance the catalytic activity. On the contrary, and for reasons not yet explained, a significant activity drop was observed, again with the singular exception of MB.

### 2.2. Monopersulfate

MPS, as a very strong oxidant, was capable of bleaching all the tested dyes regardless of the presence of the studied catalyst. However, some differences were observed: in general, catalyst efficiency decreases as pH increases, and, in particular, MB was the worst substrate, especially at higher pH. However, it is worth noting that MPS in the absence of any catalyst became more and more efficient as pH rises (see on Supplementary Figure S6). On the whole, the results obtained with this oxidant were difficult to rationalize, as the experimental errors were rather high. This is due to the strong oxidizing power of MPS, which is capable of significantly attacking the studied dyes even in the absence of any catalyst. In addition, the differences, when present, between acetylated and non-acetylated support remain unexplainable at the moment.

### 2.3. Enzymes

The biomimetic bleaching was also compared with enzymatic catalysis, using common oxidases such as laccase and peroxidases (including ligninolytic peroxidases), because these enzymes are involved in the oxidation of similar aromatic (phenolic and non-phenolic) substrates [12,17,42,43]. The results are encompassed in Table 1.

On the whole, thionines were scarcely sensitive to these enzymes. Bleaching was limited and proceeded slowly. Besides this, the substrate specificity of each enzyme was quite narrow compared with the ability of immobilized FeTPFPP to bleach all the tested thionines [44]. This strongly indicates potential applications of the described biomimetic catalysts in the industrial treatment of complex mixtures of dyes (for example, deriving from a textile factory). The UV–Vis absorption spectral changes of the thionine dyes upon treatment with the enzymes in the presence of H_2_O_2_ are shown in the Supplementary Materials Section (Figures S1–S4).

### 2.4. Multicyclic Assays

FSG-Pyr/FeTPFPP was also able to perform multicyclic bleaching of the studied dyes, as shown in Table 2. Negligible differences can be observed between the dyes. Hydrogen peroxide was a quite aggressive oxidant, leading to the loss of catalytic activity after 5–6 cycles. On the contrary, MPS was able to preserve the main part of catalytic activity for up to 10 cycles. The observed behavior of the two oxidants is easily explained by the high tendency of the *Cpd 0* formed by H_2_O_2_ to undergo hemolytic cleavage, affording the destructive hydroxyl radical.

## 3. Discussion

A certain weakness of the C–N bonds is a noticeable feature in methylated thionines In fact, when they are converted into the corresponding non-ionized bases under alkaline conditions, a slow but measurable hydrolysis takes place, releasing methanol and leading gradually from MB to TIO [7]. The change is evident, as the hypsochromic effect of demethylation produces a color shift from deep blue to violet. In a previous study [20], we have shown that such a demethylation took place when AZB was bleached with H_2_O_2_ in the presence of a tetrakis-5,10,15,20(2,6-dichlorophenyl-porphyrin manganese^III^ (MnTDCPP)-based heterogeneous catalyst, bearing pyridyl functions as the ligands for the metalloporphyrin. In that case, a sharp hypsochromic effect was seen in the long-wave region of the spectral range. This indicated the gradual *N*-demethylation of MB along the bleaching process.

**Figure 9:**
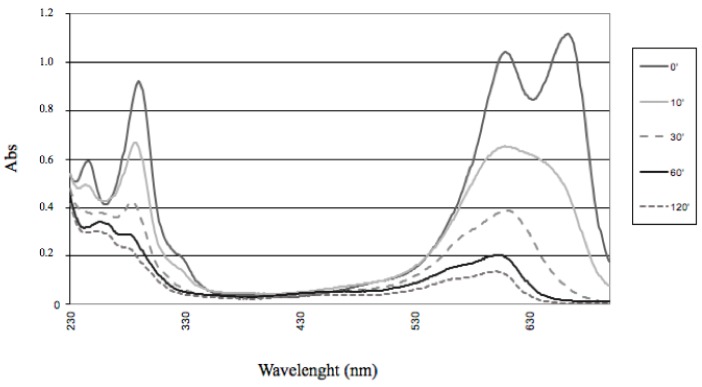
Spectral changes during MB oxidation by FSG-Pyr/FeTPFPP in the presence of hydrogen peroxide.

In the case of the present study, a different bleaching pattern was observed (Figure 9).

This could be reasonably explained by considering the different properties of Fe-porphyrins, compared with their Mn-containing counterparts [45,46]. Only a modest and ill-defined hypsochromic shift was observed along *N*-methylated thionine bleaching, giving a ‘selective’ destruction of the peak, corresponding to the highest wavelength. By waiting for a reasonable time, all the visible parts of the spectrum disappeared, while only a moderate absorption survived in the UV region. Therefore, no thionols and/or thionolins, earlier identified as products of MB oxidation under different experimental conditions [47], were formed as stable degradation products, because such compounds (with oxo- and hydroxy-functionalities replacing one or both of the original amine functionalities) are highly colored. A certain extent of oxidative *N*-demethylation, followed or accompanied by the oxidation of the sulfur atom, therefore leading to the break of the electron delocalization along the thionine nucleus, could be suggested. With respect to the ability of ligninolyitic enzymes to perform oxidation of thionines, Ferreira-Leitão et al. [44] have noted feeble activity by horseradish peroxidase (HRP) against MB, AZB, and AZA, whereas AZC was inert enough to accumulate in time into the reaction medium. Under the experimental conditions described here, a feeble but recordable activity was found, increasing from TIO to MB. In particular, TIO and AZC remained practically unchanged (therefore confirming the finding of Oliveira et al. [32] about the inertness of AZC and its accumulation into the reaction mixture). As noted above, MB was a substrate for the enzyme. However, no hypsochromic shifts were observed, suggesting a non-demethylating mechanism for the dye bleaching (see the Supplementary Materials).

In the case of the chosen fungal laccase (from *Pleurotus pulmonarius,* previously known as *P. sajor-caju* [48]), practically no activity was observed after 3 h incubation (not shown). This finding apparently contradicts the results of Forootanfar et al. [9], who found that at least one fungal laccase was able to bleach MB. It is worth noting that two laccases used by Forootanfar et al. were also practically inactive, and that the phenothiazine ring *per se* is not inert toward the action of fungal laccases [49]; thionine dyes, however, contain the phenothiazinium cation, which is conceivably much more resistant against oxidation by the enzyme.

Lignin peroxidase (LiP, from *Phanerochaete chrysosporium*) has been reported as able to bleach thionine dyes [50], so AZB was proposed as a selective LiP substrate, provided that it is inert toward other common enzymes usually accompanying LiP, such as laccase and manganese peroxidase (MnP). Our experiments confirmed that finding, but also showed that MB is the best substrate for the enzyme, whereas AZA was a poorer substrate, and both TIO and AZC are quite inert under the studied conditions.

Quite singular and not easily understandable was the behavior of the thionine dyes under catalysis by MnP in the presence of H_2_O_2_ and Mn^II^ acetate, as detailed in the experimental Section. First of all, TIO remained nearly unchanged upon enzyme action, whereas the other dyes showed a typical spectral modification. Such a modification did not affect the visible part of the spectrum (contrary to that observed for all the catalysts under these study conditions, both chemical and enzymatic) but provoked a transient absorbance increase of the peak in the near UV region within the first ten minutes, followed by a sharp decrease. The lack of any modification in the long-wave region of the spectrum strongly indicates that neither demethylation nor chromophore breaking take place; however, the mechanism of the chemical modification is waiting for a rational explanation. The only conclusion one may draw from the experiments is that the presence of at least one methyl substituent on the peripheral nitrogen atoms is mandatory for a thionine dye to behave as a substrate. The described behavior could be compared to that described for the chemical oxidation of the thionine dyes catalyzed by Mn^III^ sulfate dissolved in concentrated (2 to 5 M) sulfuric acid [8]. In that study, the formation of detectable colored intermediates, such as methyl thionoline, thionoline, and thionol, was observed. However, under the strongly acidic conditions those Authors have adopted, the degradation reactions took place on the thionine dications, owing to the protonation of the ring nitrogen. Therefore, the different degradation patterns and different reaction intermediates/products are the almost obvious result of quite different experimental starting conditions.

## 4. Materials and Methods

All the reagents were of the best grade available, and were used without any further purification. The sole exception was fungal laccase (LC), and LC was produced and purified as already described [51] UV–Vis spectra and absorbance readings were recorded using an Ultrospec 2100pro UV–VIS spectrophotometer (Amersham Biosciences, Milan, Italy).

FT-IR spectra were recorded after the samples were prepared as KBr pellets, with a KBr beam-splitter and KBr windows using a Thermo Nicolet 5700 (Waltham, MA, USA) spectrometer at room temperature.

Sample morphology was observed using scanning electron microscopy (SEM) (S-4100, Hitachi, Tokyo, Japan). The samples were fixed on a brass stub using double-sided carbon coated with gold blazers on a SCD 004 sputter coater (Hitachi, Tokyo, Japan) for 2 min and observed under an excitation voltage of 5 kV.

### 4.1. Supports

A silica-based support was prepared from fumed silica as follows: 10 g of fumed silica was suspended in water to form a free-flowing slurry. The fines were removed by repeated decantation steps. The slurry was then filtered, and the obtained wet paste repeatedly washed with 2-propanol and finally dried in an oven at 80 °C. 

The dried fumed silica was treated in a glass flask with a 20% solution of 2[2(3-trimethoxysilylpropylamino)ethylamino]ethylamine, alias 3[2(2-aminoethylamino)ethylamino]-propyltrimethoxysilane, in diethylene glycol dimethyl ether. The slurry was placed in an oven at 80 °C overnight.

The silanized silica was washed successively with 0.1 M HCl, water, 0.1 M NaOH, and finally again with water to neutrality. The wet paste was recovered by suction filtration, dehydrated by 2-propanol washings, and finally dried in an oven at 80 °C.

The product was treated with excess 10% pyridine-4-carboxyaldehyde solution in diethylene glycol dimethyl ether. The suspension was gently stirred for 20 min and then treated dropwise with excess sodium cyanoborohydride in aqueous 0.1 M NaOH, and stirred for a further 12 h. The product was washed with 2-propanol and water. The wet paste was filtered by suction, washed again with 2-propanol, and finally dried in an oven at 80 °C.

For certain experiments, the obtained product was subjected to a further treatment, to achieve acetylation of the –NH– bridges along the silane chain. For this purpose, the dry support (200 mg) was suspended in diethylene glycol dimethyl ether (4 mL), then 400 μL of *N*-methylmorpholine and 800 μL of acetic anhydride were added. The suspension was stirred overnight, and then washed with isopropanol followed by water until neutral, and finally dried again in an oven at 80 °C.

For the synthesis of imidazole-grafted supports, imidazole-4(5)-carboxyaldehyde was substituted for pyridine-4-carboxaldeyde as the alkylating agent for the primary amino groups of the silane tethers. The reduction protocol was identical to that described above.

### 4.2. Heterogenized Catalysts

The metalloporphyrin (12 mg of FeTPFPP) was dissolved in 1.5 mL of DMSO, and the dark brown solution was mixed with 600 mg of the chosen functionalized fumed silica. The obtained slurry was slowly inverted end-over-end overnight, and then repeatedly washed with isopropanol until no more metalloporphyrin was extracted. The brown-blackish product was vacuum filtered and dried at 80 °C in an oven. The obtained catalyst was stored in the dark at room temperature until used. In this way, two different catalysts were obtained, immobilized on pyridyl-functionalized fumed silica, with or without previous acetylation of the support. For certain experiments, two other catalysts were also prepared, bearing the imidazole functionalization as the ligand for the iron center of the porphyrin. All the putative structures of the four synthesized supports are shown in Figure 5.

### 4.3. Support Characterization

Particle morphology was checked by means of scanning electron microscopy. FT-IR spectrometry was used to assess the functionalization of the supports.

Functionalization degree was determined through total nitrogen analysis, as already described [20]. Potassium peroxydisulfate was used as the oxidant (46.5 g·L^−1^) in the presence of 15 g·L^−1^ boric acid, and 7 g·L^−1^ sodium hydroxide. Samples (2 mg) were suspended in 2.5 mL H_2_O and 0.7 mL oxidant solution. After 30 min at 120 °C, the supernatant was treated with 15 μL of 98% H_2_SO_4_, and absorbance at 220 nm was recorded.

### 4.4. Catalytic Assays

The five phenothiazine dyes, namely TIO, AZC, AZA, AZB, and MB, belonging to the homologous series of *N*-methylated thionine derivatives, were studied as the substrates for the heterogenized catalysts. Two oxidizing agents were tested, i.e., hydrogen peroxide and potassium monopersulfate (MPS, 2KHSO_5_·KHSO_4_·K_2_SO_4_). The oxidation was measured by UV–VIS spectroscopy, assessing the percentage of decolorization, at the wavelength of maximum absorption (in the visible range) for each dye at pH 3: λ = 603 (48,000 M^−1^·cm^−1^), 619 (35,000 M^−1^·cm^−1^), 633 (55,000 M^−1^·cm^−1^), 648 (61,000 M^−1^·cm^−1^), 667 (74,000 M^−1^·cm^−1^) nm for TIO, AZC, AZA, AZB, and MB, respectively [52].

In order to better evaluate the catalytic activity in the presence of the two oxidants hydrogen peroxide and MPS, some blanks were prepared excluding the oxidant or the catalyst.

The samples were analyzed at predetermined times. Furthermore, all the tests were carried out at different pH values using 50 mM McIlvaine buffers at pH 3, 5, and 7, respectively.

Decolorization was quantified at 25 °C by spectrophotometric assay: 5 mg of catalyst was poured—in a final volume of 1 mL containing 25 mM buffer, 8.8 mM H_2_O_2_ or MPS, and 15 mM of the chosen dye—into a glass test tube, which was maintained under constant stirring in an orbital apparatus. At fixed times, the stirring was stopped, 1 mL of 1 M citrate buffer (pH 3) was added, and the supernatant was used to measure absorbance variation. Catalytic activity was expressed as % decolorization, i.e., the % of initial absorbance at λ_max_ which disappeared during reaction.

### 4.5. Enzymes

Some enzymes were used: laccase (EC 1.10.3.2) from *Pleurotus pulmonarius* (also formerly known as *P. sajor-caju* [51]), horseradish peroxidase (HRP, EC 1.11.1.7, Sigma-Aldrich, Milan, Italy) from *Armoracia rusticana*, lignin peroxidase (LiP, EC 1.11.1.14, Sigma-Aldrich) from *Phanerochaete chrysosporium*, and manganese peroxidase (MnP, EC 1.11.1.13, Sigma-Aldrich) from *Ph. chrysosporium*. When these enzymes were used, the composition of assay mixtures were changed where appropriate with the systematic omission of the biomimetic catalyst as follows: laccase, 23 E.U., 50 mM potassium phosphate buffer (pH 6); HRP, 3 E.U., 50 mM potassium phosphate buffer (pH 7), 8.8 mM H_2_O_2_; LiP, 0.05 E.U., 50 mM sodium citrate buffer (pH 3), 0.176 mM H_2_O_2_; MnP, 0.02 E.U., 50 mM sodium citrate buffer (pH 3), 0.176 mM H_2_O_2_, 2mM Mn(II) acetate, dissolved in 50 mM sodium malonate buffer (pH 4.5).

## 5. Conclusions

This paper reported the first complete screening of substituted thionine oxidation by immobilized metalloporphyrins, emulating lignin peroxidase. H_2_O_2_ alone was, on the whole, a quite ineffective oxidant for thionines, regardless of the *N*-methylation degree, but noticeable oxidation took place in the presence of the described catalysts at pH 3. On the other hand, the catalyst effectiveness became quite poor at pH 5 and pH 7. By contrast, MPS alone was able to bleach all the studied thionines with noticeable efficiency. However, the oxidation became even faster in the presence of the described catalysts, at least under certain experimental conditions.

The bleaching mechanism was under study, although some points have been already ascertained: (*i*) no selective *N*-demethylation of the methylated thionines took place, contrary to that observed in a previous study using a Mn-TDCPP-based catalyst; (*ii*) no thionols and/or thionolins could be isolated as stable bleaching products, provided that the bleaching experiments always gave colorless solutions when the bioinspired catalysts were used; (*iii*) the oxidation of the sulfur atom (to sulfoxide/sulfone with possibly further oxidation leading to the breakdown of the thiazine ring) could be reasonably suggested, provided that such an oxidation must finally afford colorless products. However, the possible occurrence of an alternative mechanism has been suggested and discussed.

Further studies are in progress to gain insight on the degradation pathways of the studied thionine dyes.

## Figures and Tables

**Table 1:** Enzymatic bleaching of the tested dyes. Legend: LC laccase, HRP horseradish peroxidase, LiP lignin peroxidase, MnP manganese peroxidase; + substrate, +/− weak substrate (bleaching < 10% in 24 h), − no substrate.

Dye	LC	HRP	LiP	MnP
TIO	−	−	−	−
AzC	−	−	−	+/−
AzA	−	+/−	−	+/−
AzB	−	+/−	+/−	+/−
MB	−	+	+	+/−

**Table 2:** Multicycle use of FSG-Pyr/FeTPFPP in the presence of both MPS and hydrogen peroxide at pH 3.

Cycle	% Residual Activity			
	MPS		H_2_O_2_	
	MB	TIO	MB	TIO
**1**	100 ± 5	100 ± 2	100 ± 2	100 ± 4
**2**	86 ± 3	81 ± 6	31 ± 3	10 ± 3
**3**	86 ± 5	65 ± 4	21 ±1	8 ± 4
**4**	87 ± 6	56 ± 2	11 ± 2	7 ± 2
**5**	82 ± 3	53 ± 7	8 ± 3	8 ± 4
**6**	91 ± 4	61 ± 6	2 ± 1	9 ± 6
**7**	87 ± 5	54 ± 4	4 ± 1	4 ± 2
**8**	85 ± 7	57 ± 7	5 ± 2	5 ± 2
**9**	95 ± 8	60 ± 2	1 ± 1	8 ± 2
**10**	87 ± 4	63 ± 5	5 ± 1	9 ± 4

## Supplementary Materials

Supplementary materials can be found at www.mdpi.com/1422-0067/18/12/2553/s1.

## Abbreviations

Ac-SFG-Pyr/FeTFPP	*N*-Acetyl 4-Pyridyl 3-[2-(2-aminoethylamino)-ethylamino]-propyl-trimethoxysilane-fumed silica/5,10,15,20-tetrakis(pentafluorophenyl)porphine
AZA	Azure A
AZB	Azure B
AZC	Azure C
*Cpd*	*Compound*
ET	Electron transfer
FeTFPP	5,10,15,20-Tetrakis(pentafluorophenyl)porphine iron(III)-chloride
HAT	Hydrogen atom transfer
HRP	Horseradish peroxidase E.C. 1.11.1.7
LC	Laccase E.C. 1.10.3.2
LiP	Lignin peroxidase E.C. 1.11.1.14
MB	Methylene blue
MPS	Monopersulfate
MnP	Manganese peroxidase E.C. 1.11.1.13
OT	Oxygen transfer
SFG-Pyr/FeTFPP	4-Pyridyl-3-[2-(2-aminoethylamino)-ethylamino]-propyl-trimethoxysilane fumed silica 5,10,15,20-tetrakis(pentafluorophenyl)-porphine
SFG	Fumed silica
SFG/Pyr	4-Pyridyl-3-[2-(2-aminoethylamino)-ethylamino]-propyl-trimethoxysilane-fumed silica
TIO	Thionine
TFPP	5,10,15,20-tetrakis(pentafluorophenyl)porphine
