# Supplementary material for: Bioinspired versus Enzymatic Oxidation of Some Homologous Thionine Dyes in the Presence of Immobilized Metalloporphyrin Catalysts and Ligninolytic Enzymes
Source: Int J Mol Sci. 2017 Nov 28;18(12):2553. doi: 10.3390/ijms18122553 (PMC5751156; doi:10.3390/ijms18122553)

Figure S1. Spectral changes during horseradish peroxidase oxidation of TIO; AZC, AZA, AZB, and MB

TIO

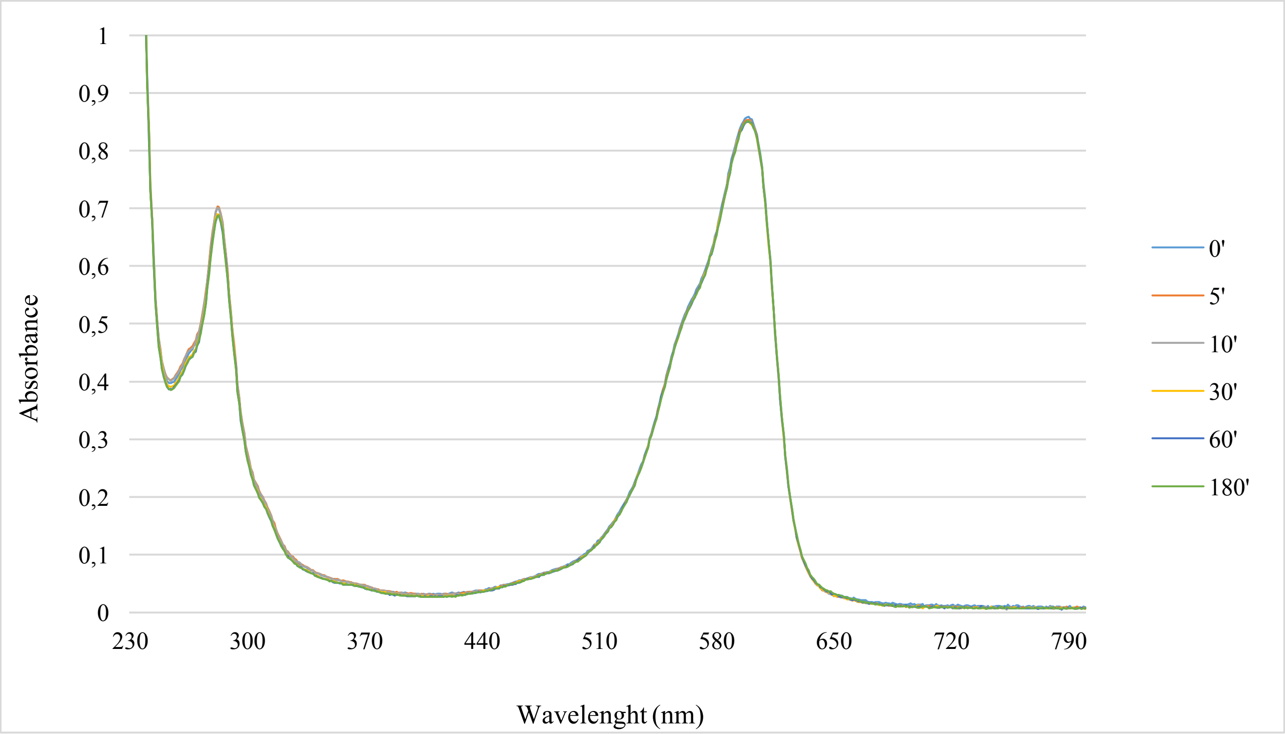

AZC

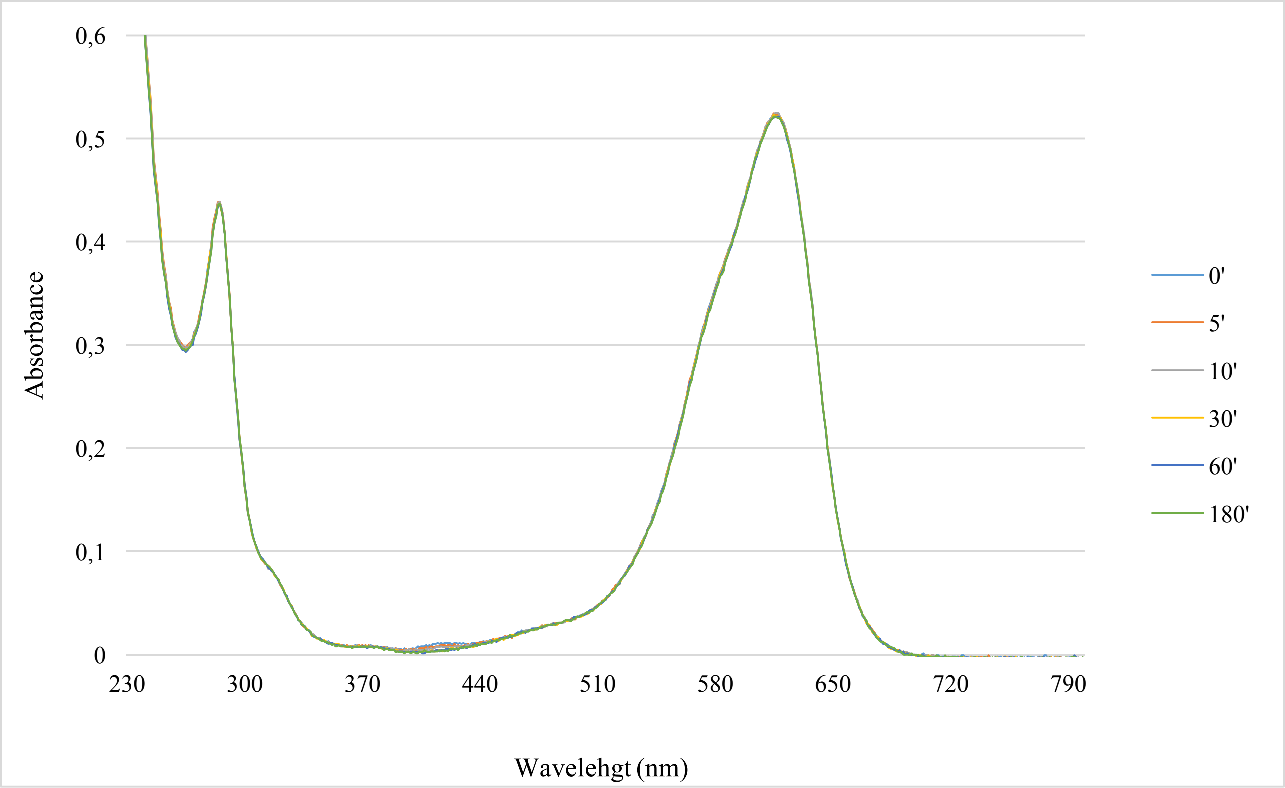

AZA

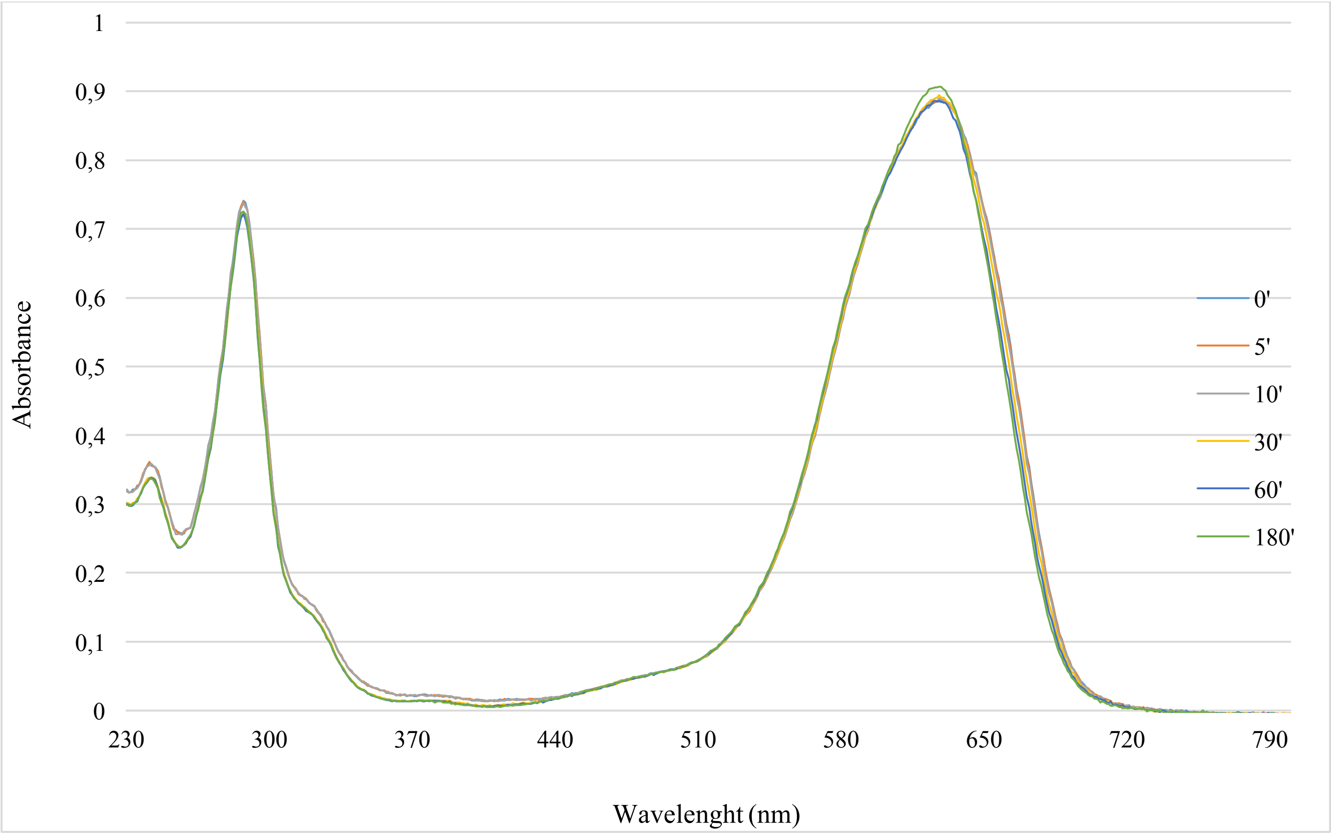

1
0,9
0,8
0,7
0,6
0,5
0,4
0,3
0,2
0,1
0
Absorbance
230
300
370
440
510
580
650
720
790
Wavelenght (nm)
0'
5'
10'
30'
60'
180'

AZB

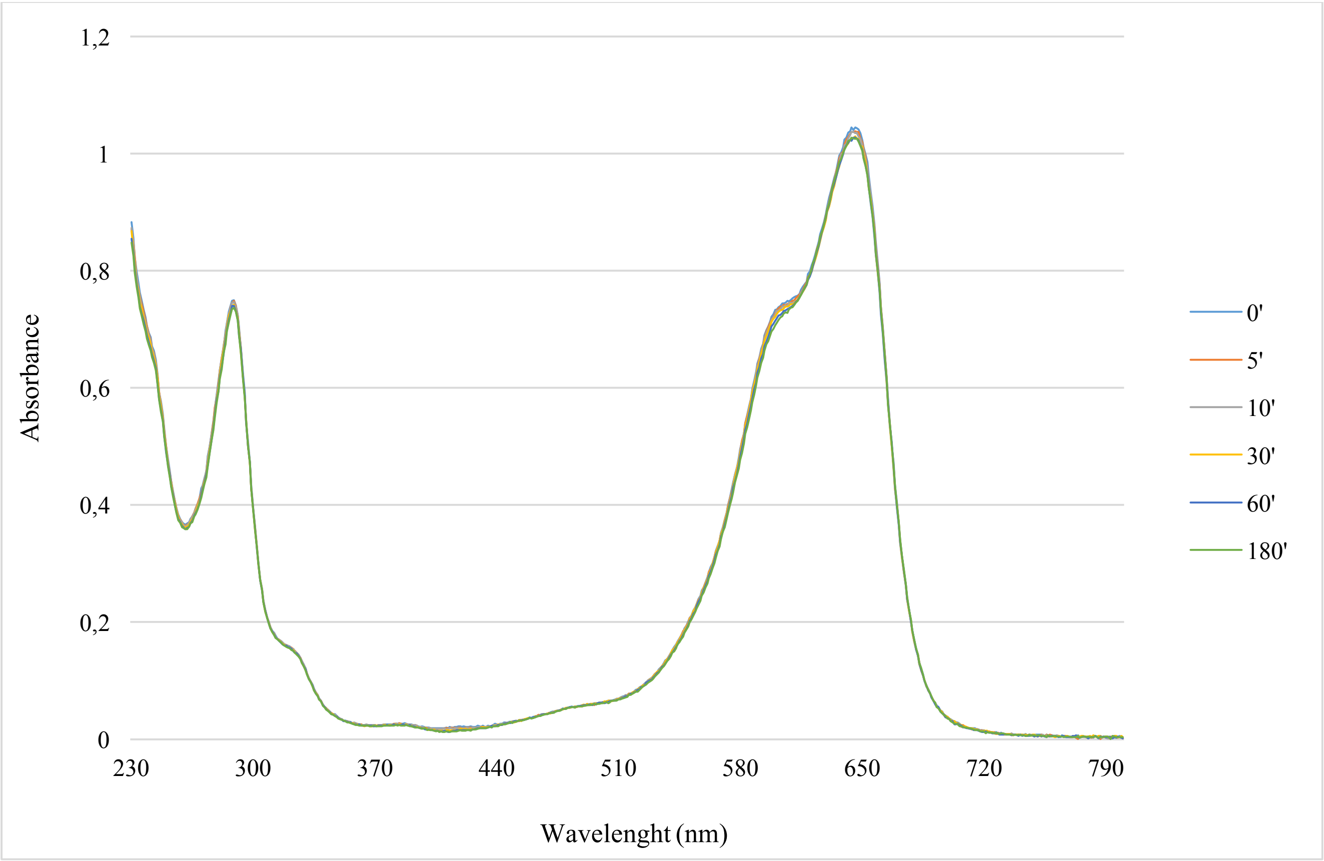

1,2
1
0,8
0,6
0,4
0,2
0
Absorbance
230
300
370
440
510
580
650
720
790
Wavelenght (nm)
0'
5'
10'
30'
60'
180'

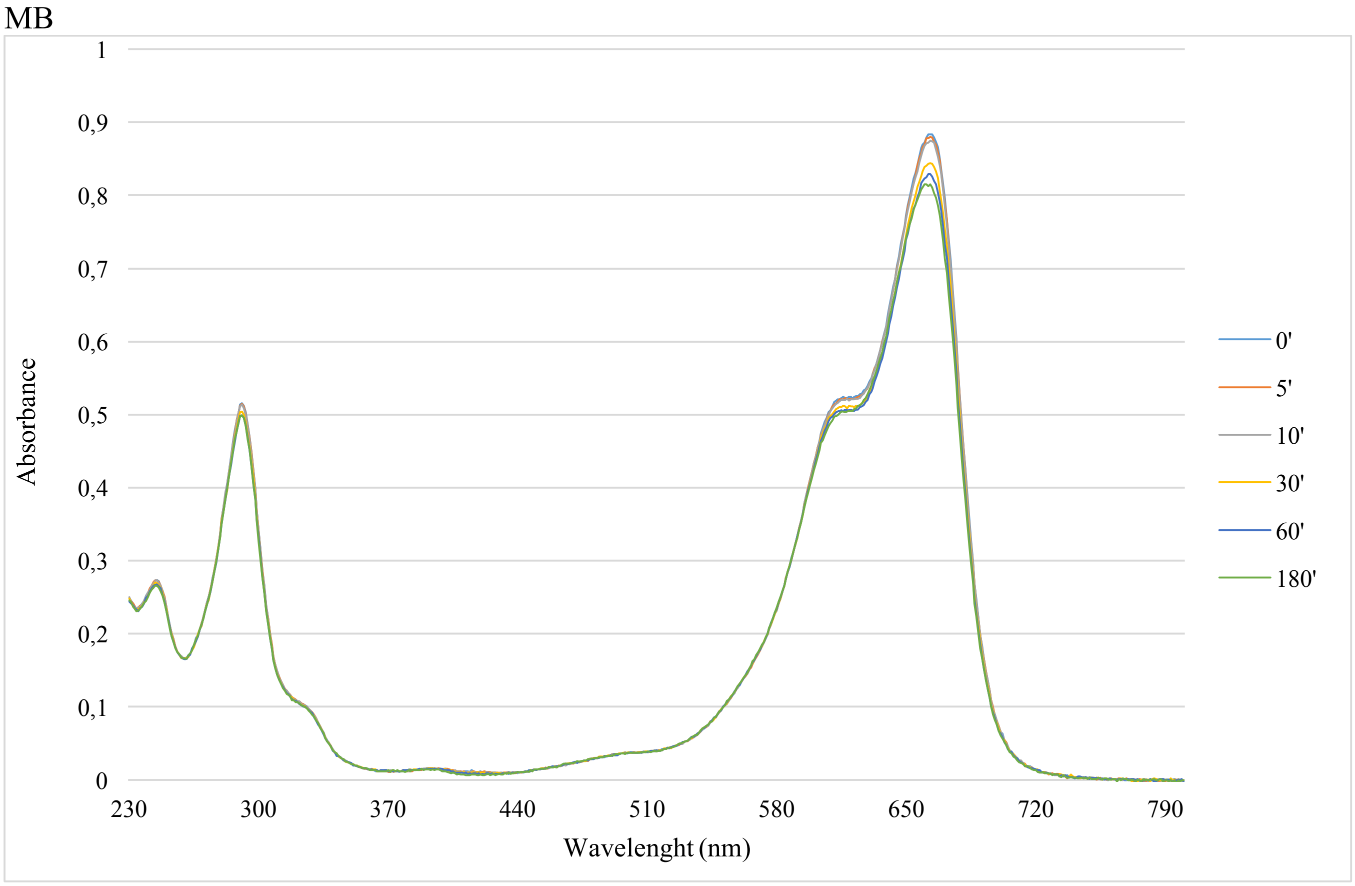

MB
Absorbance
1
0,9
0,8
0,7
0,6
0,5
0,4
0,3
0,2
0,1
0
230
300
370
440
510
580
650
720
790
Wavelenght (nm)
0'
5'
10'
30'
60'
180'

Figure S2. Spectral changes during laccase oxidation of TIO; AZC, AZA, AZB, and

MB TIO

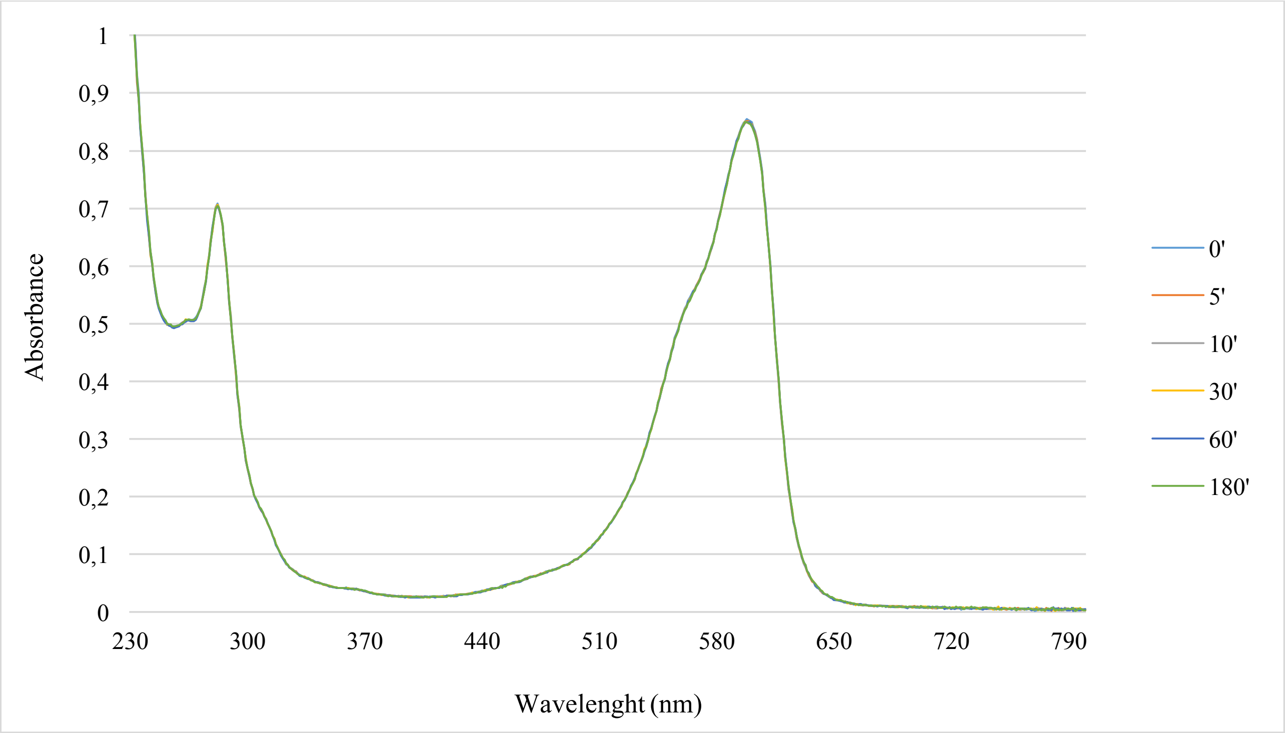

AZC

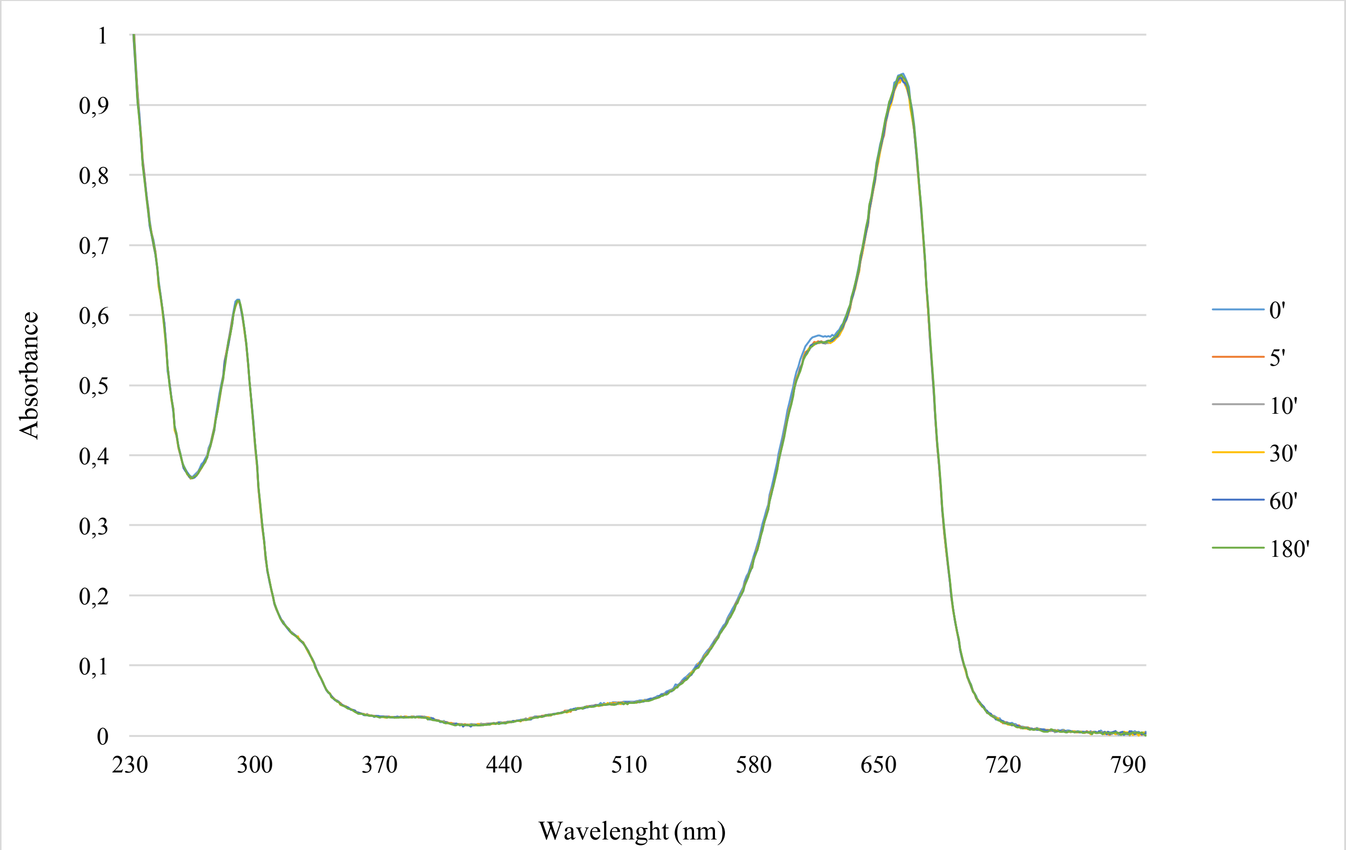

AZA

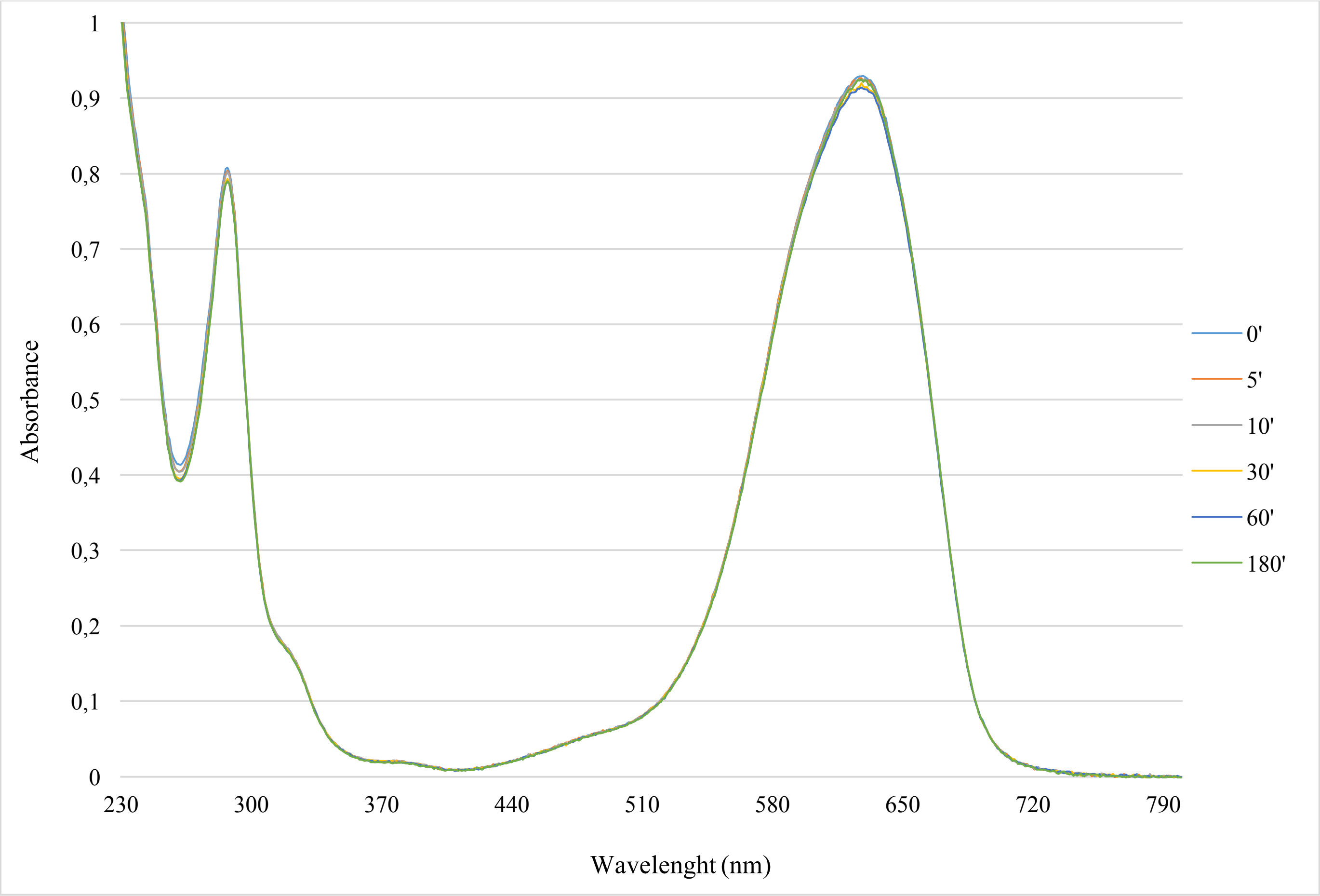
1
0,9
0,8
0,7
0,6
0,5
0,4
0,3
0,2
0,1
0
Absorbance
230
300
370
440
510
580
650
720
790
Wavelenght (nm)
0'
5'
10'
30'
60'
180'

AZB

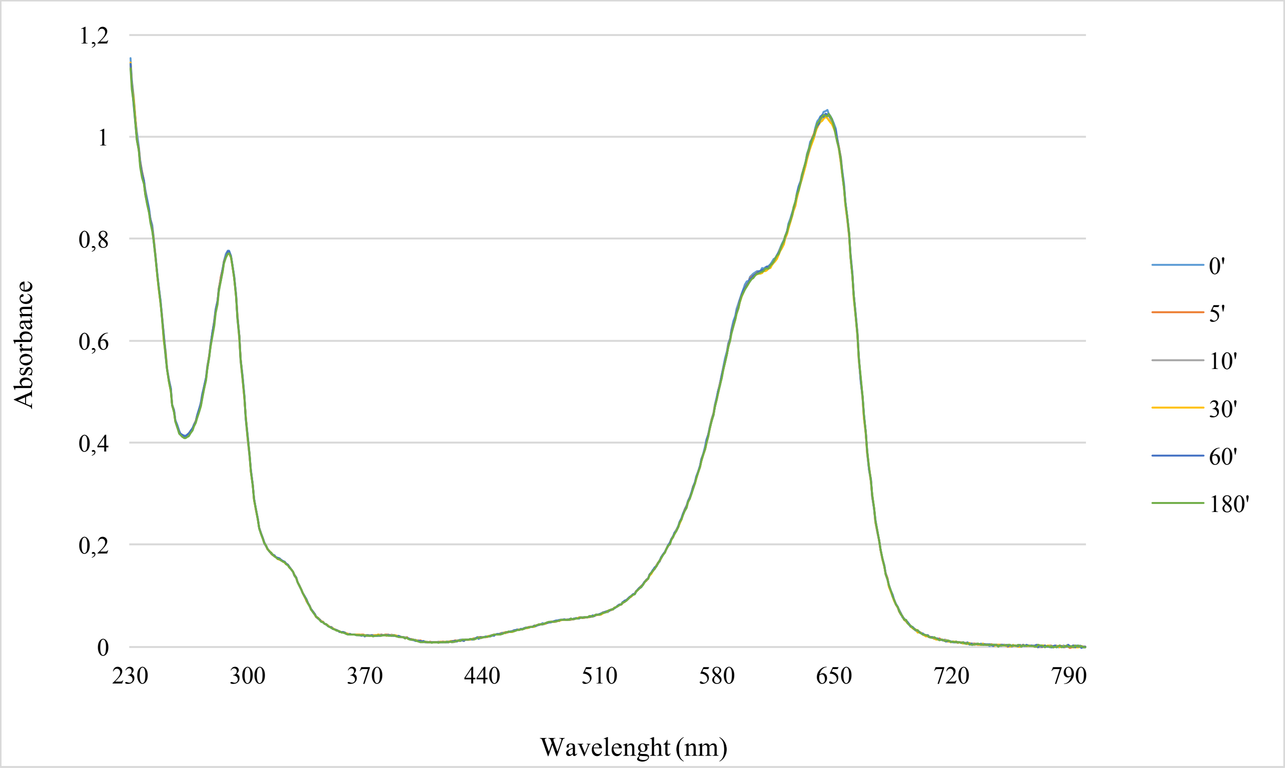
1,2
1
0,8
0,6
0,4
0,2
0
Absorbance
230
300
370
440
510
580
650
720
790
Wavelenght (nm)
0'
5'
10'
30'
60'
180'

MB

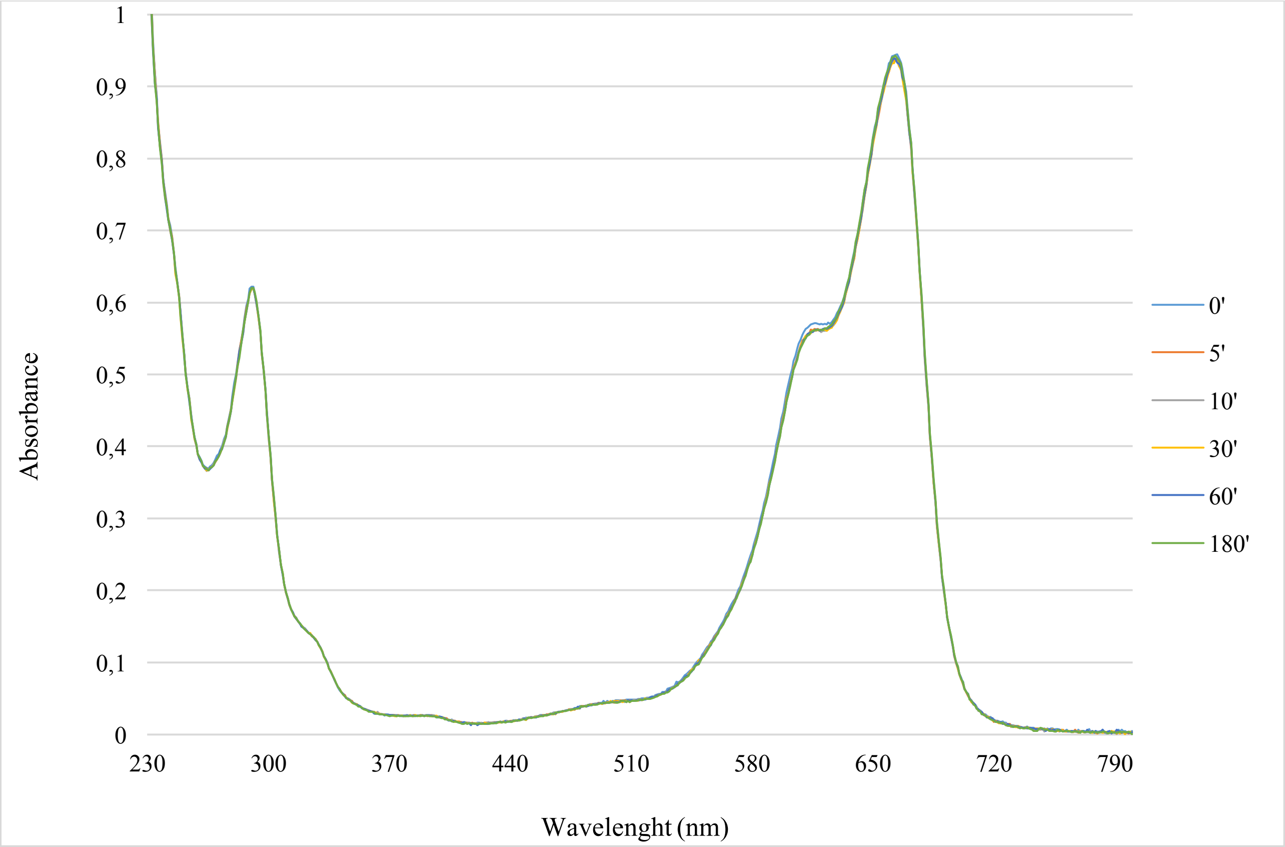
1
0,9
0,8
0,7
0,6
0,5
0,4
0,3
0,2
0,1
0
Absorbance
230
300
370
440
510
580
650
720
790
Wavelenght (nm)
0'
5'
10'
30'
60'
180'

Figure S3. Spectral changes during lignin peroxidase oxidation of TIO; AZC, AZA, AZB, and MB TIO

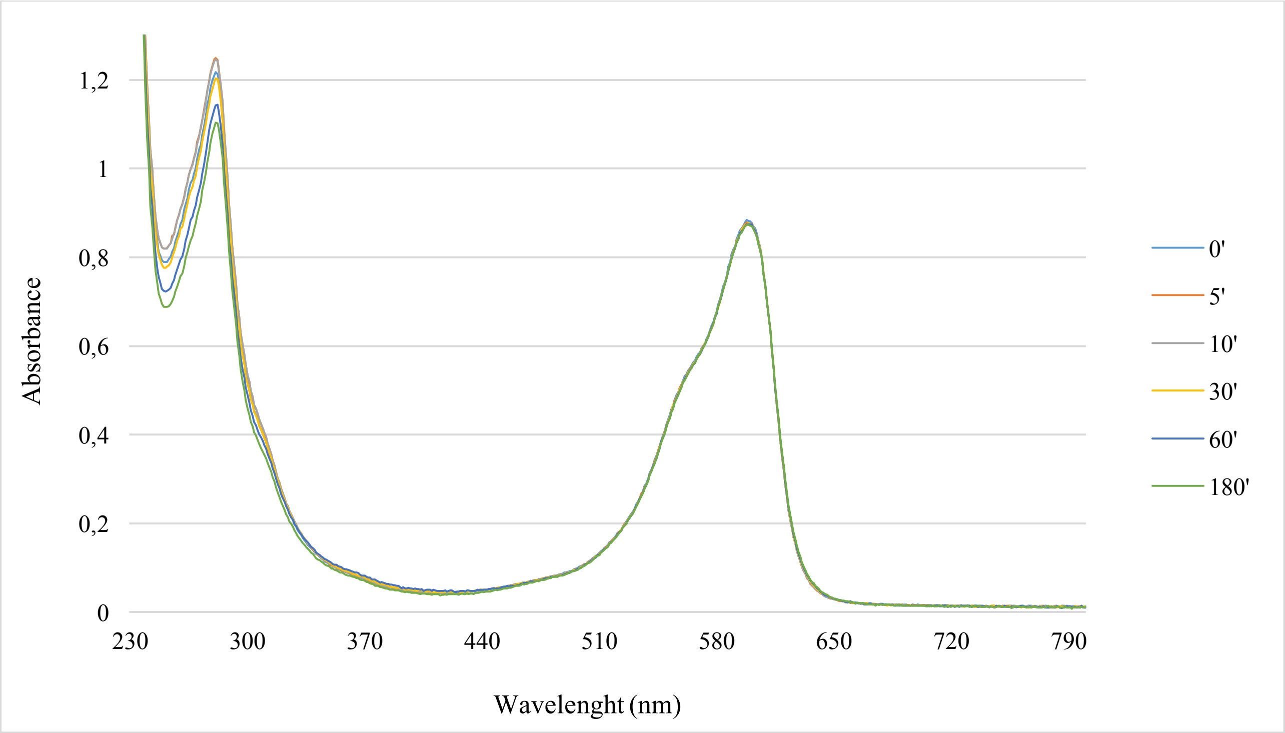

AZC

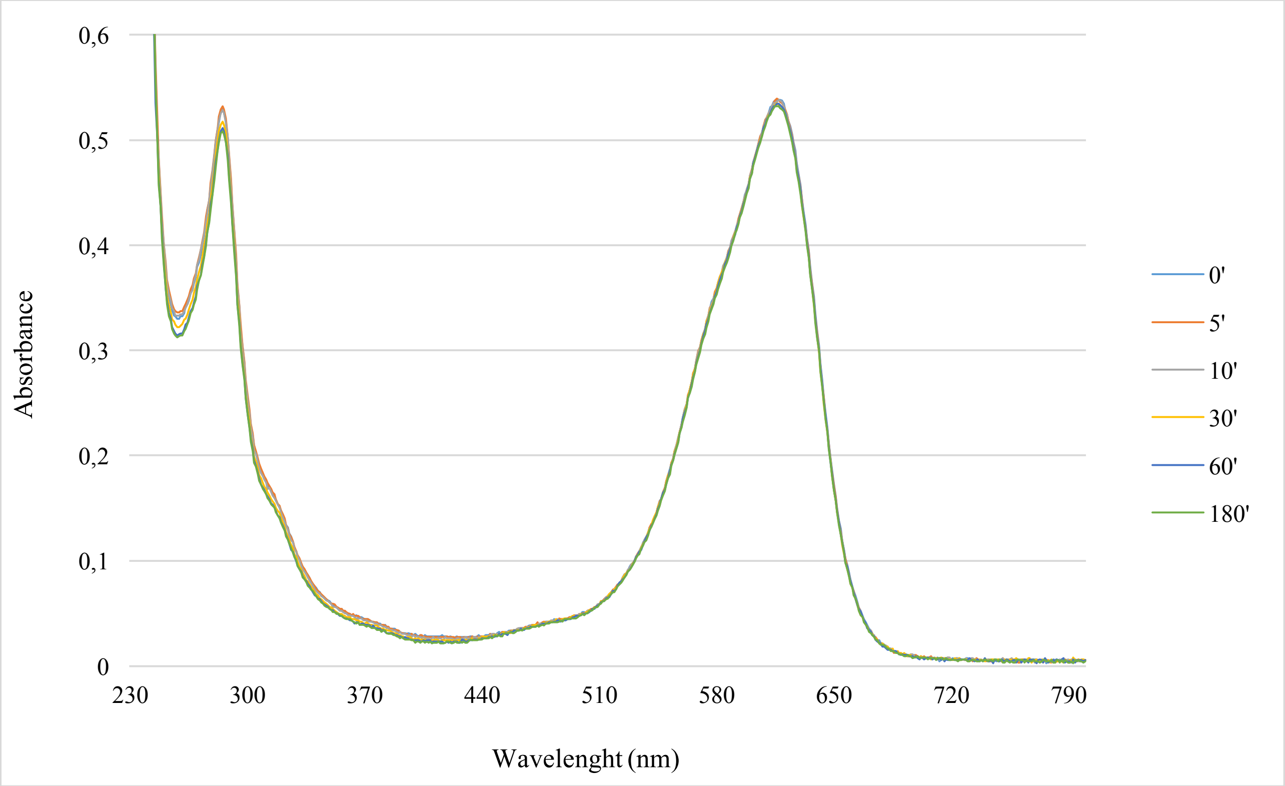

AZA

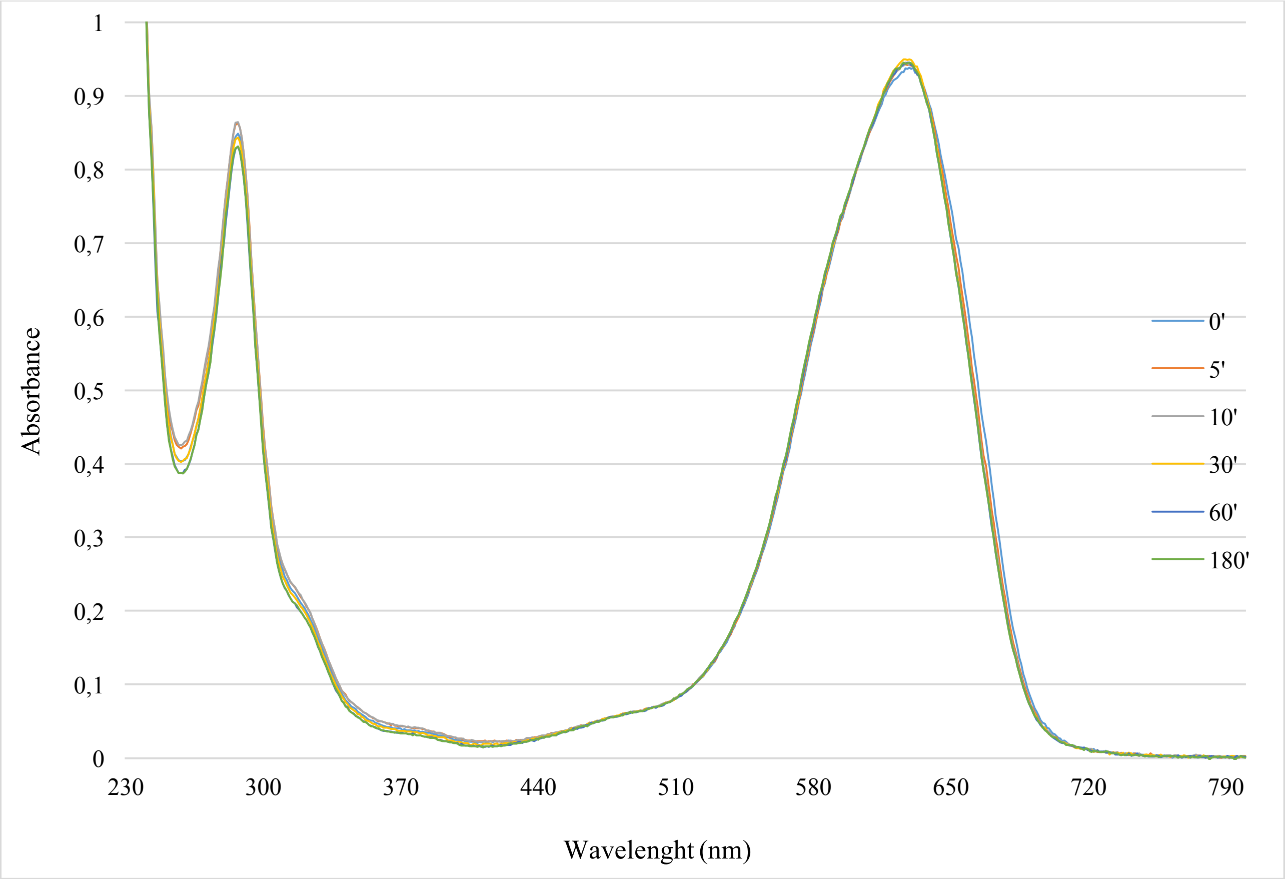
1
0,9
0,8
0,7
0,6
0,5
0,4
0,3
0,2
0,1
0
Absorbance
230
300
370
440
510
580
650
720
790
Wavelenght (nm)
0'
5'
10'
30'
60'
180'

AZB

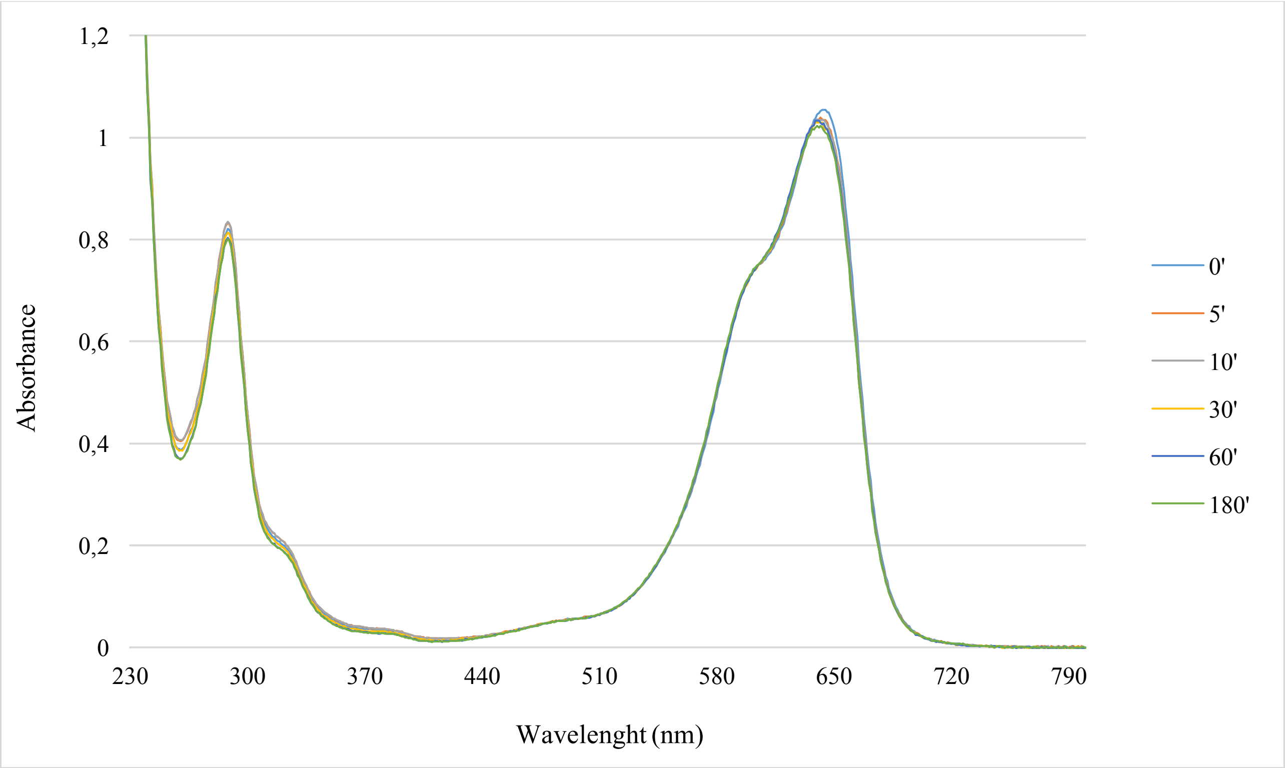
1,2
1
0,8
0,6
0,4
0,2
0
Absorbance
230
300
370
440
510
580
650
720
790
Wavelenght (nm)
0'
5'
10'
30'
60'
180'

MB

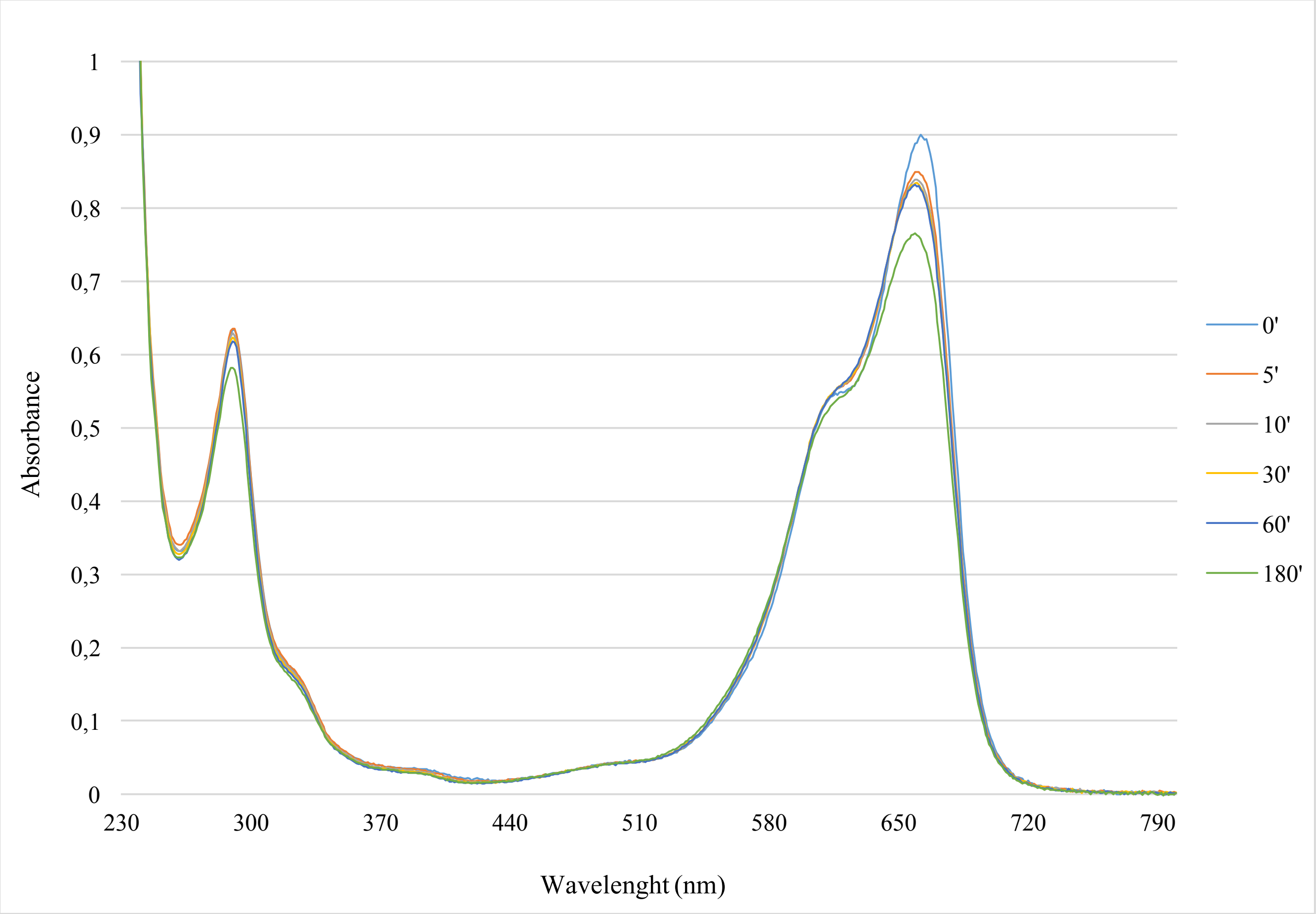

Absorbance
1
0,9
0,8
0,7
0,6
0,5
0,4
0,3
0,2
0,1
0
230
300
370
440
510
580
650
720
790
Wavelenght (nm)
0'
5'
10'
30'
60'
180'

Figure S4. Spectral changes during manganese peroxidase oxidation of TIO; AZC, AZA, AZB, and MB TIO

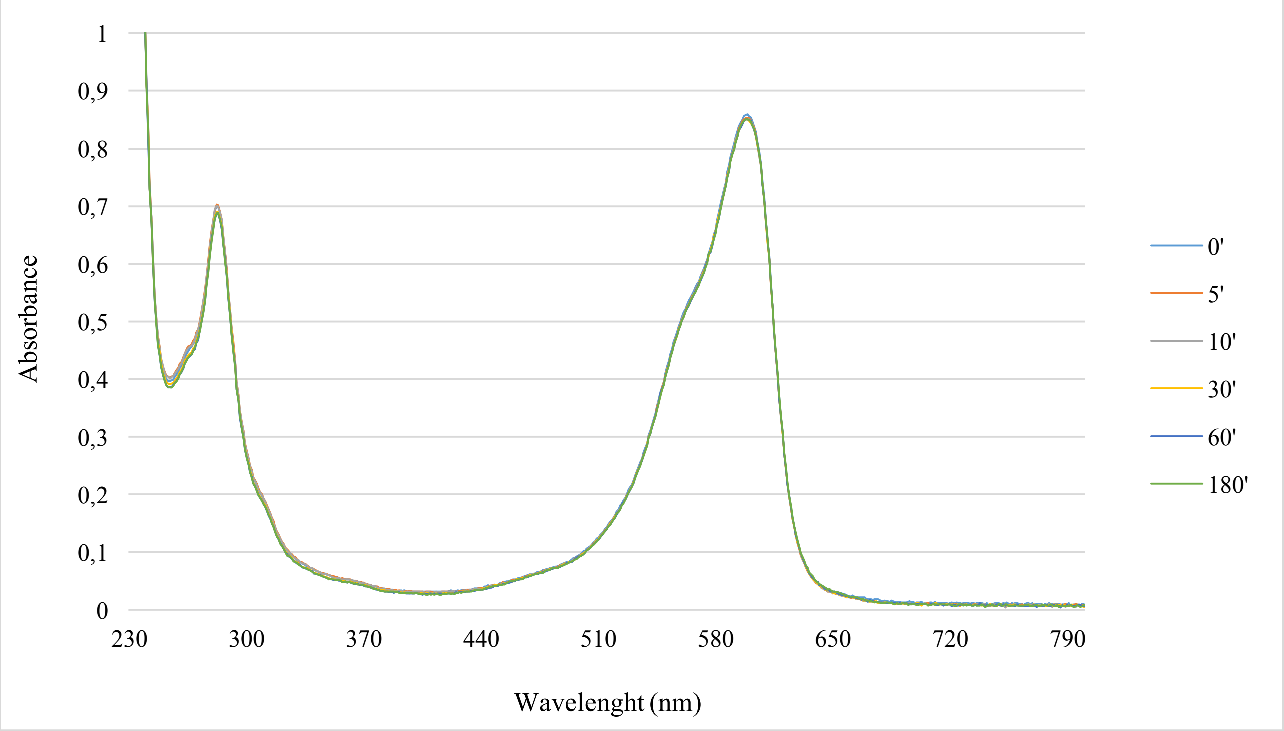

AZC

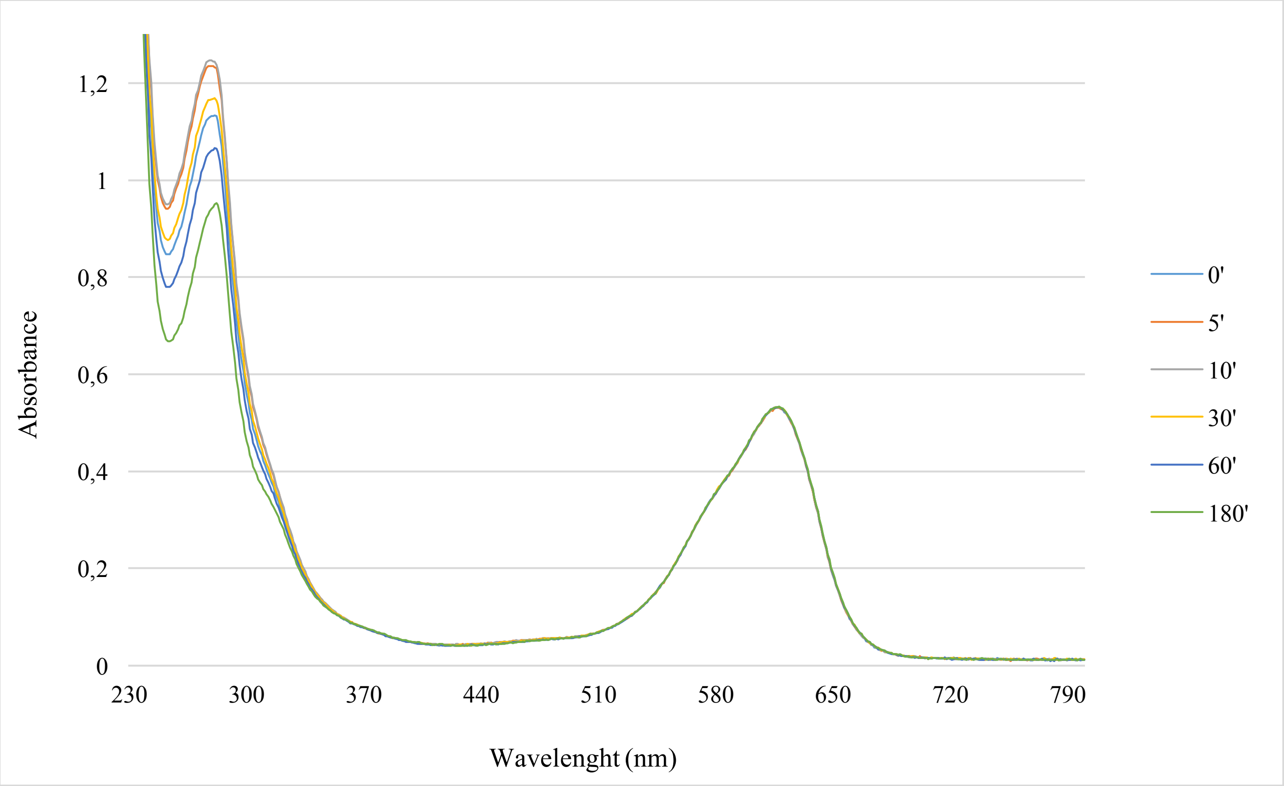

AZA

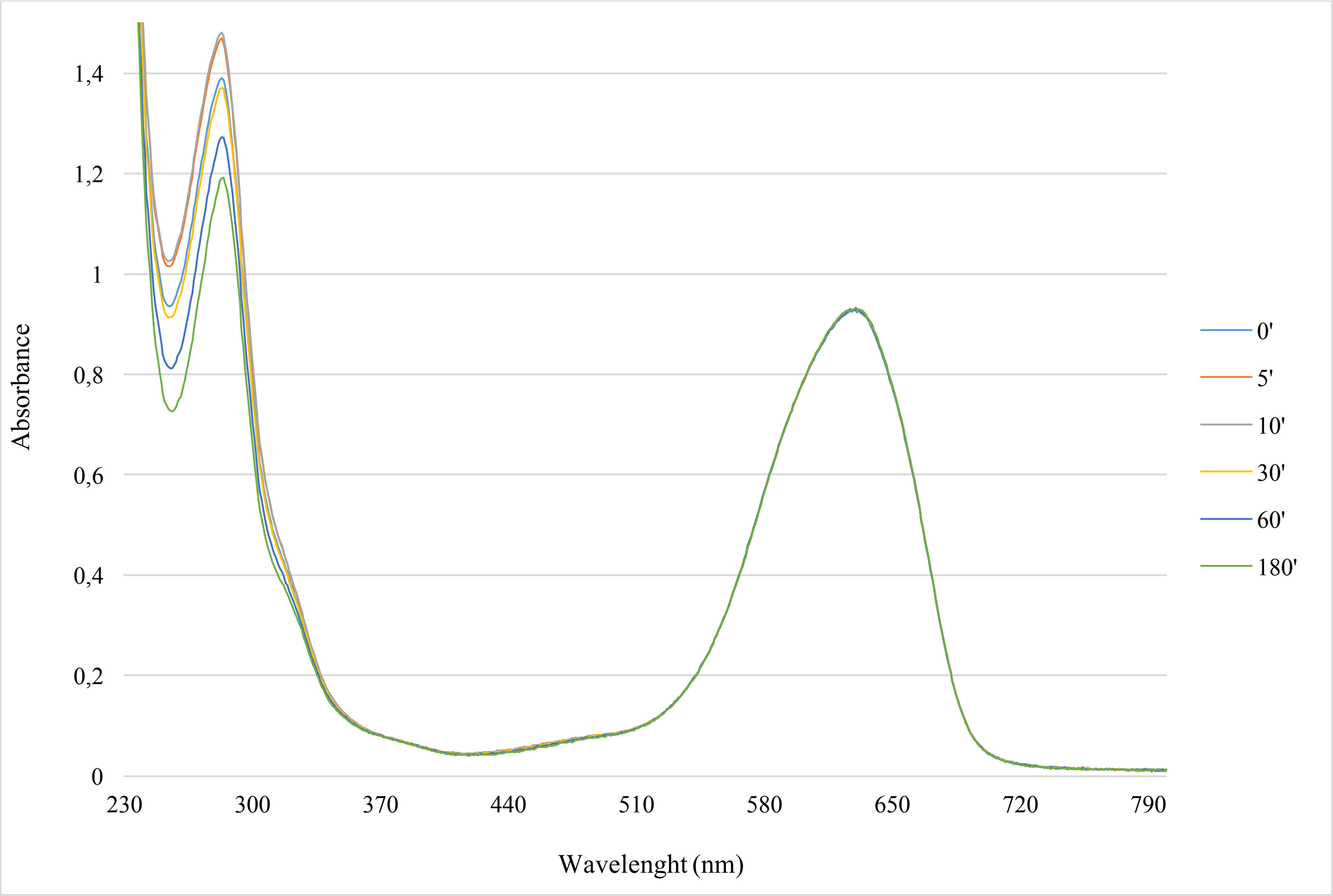
Absorbance
1,4
1,2
1
0,8
0,6
0,4
0,2
0
230
300
370
440
510
580
650
720
790
Wavelenght (nm)
0'
5'
10'
30'
60'
180'

AZB

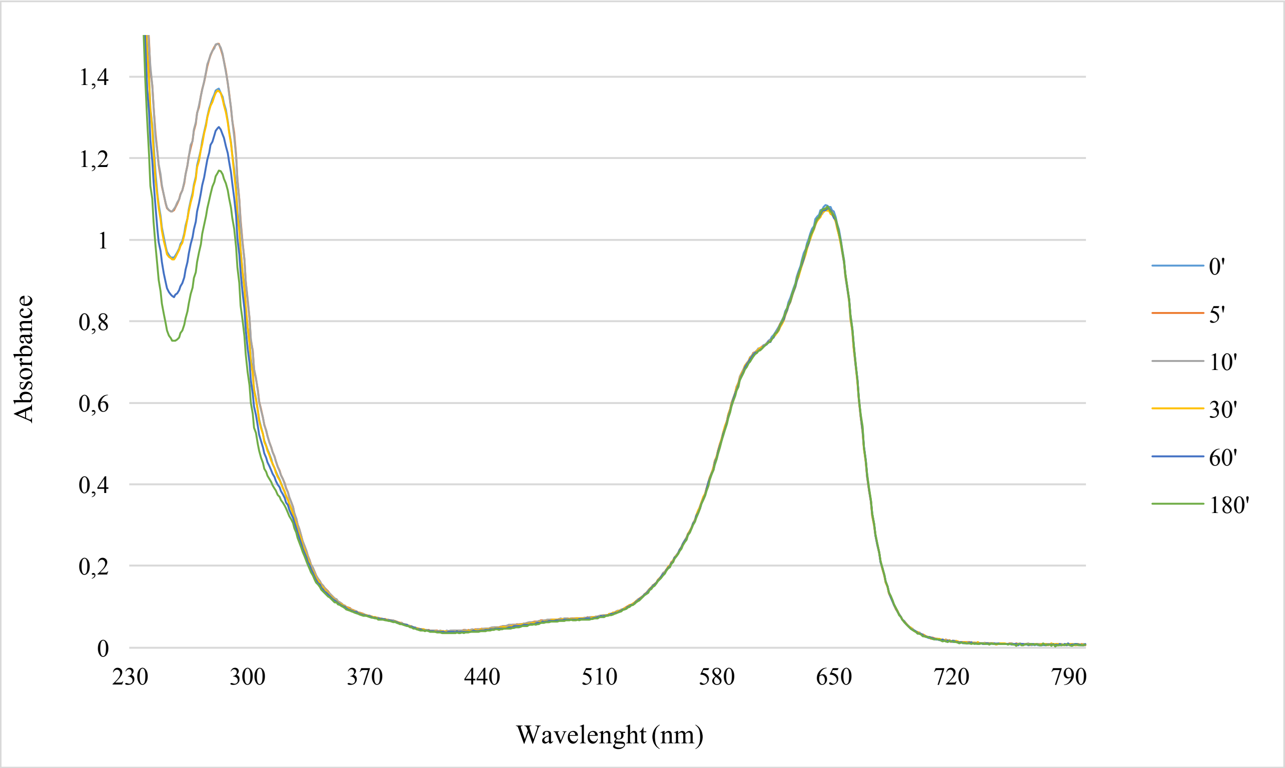
Absorbance
1,4
1,2
1
0,8
0,6
0,4
0,2
0
230
300
370
440
510
580
650
720
790
Wavelenght (nm)
0'
5'
10'
30'
60'
180'

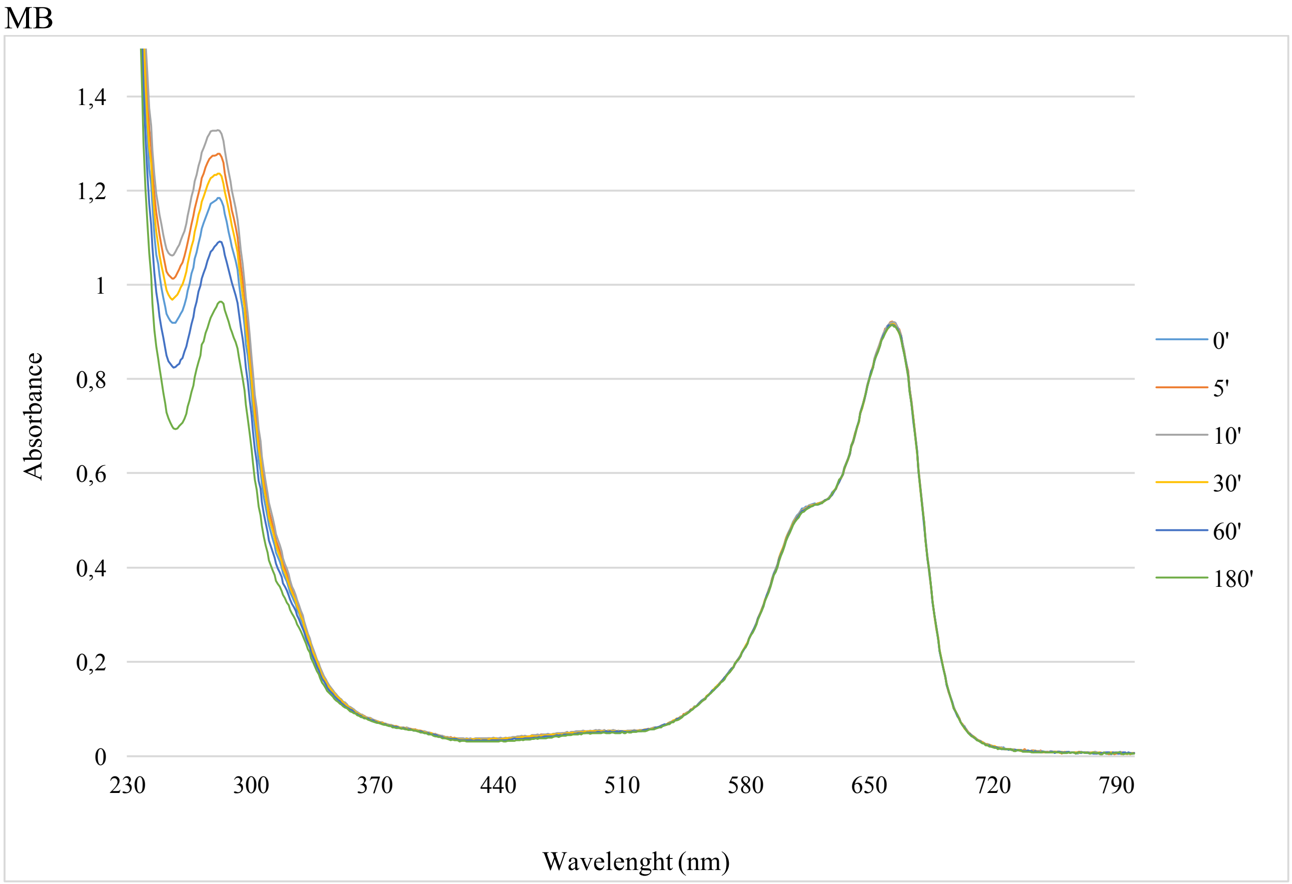
MB
Absorbance
1,4
1,2
1
0,8
0,6
0,4
0,2
0
230
300
370
440
510
580
650
720
790
Wavelenght (nm)
0'
5'
10'
30'
60'
180'

Figure S5. FT-IR spectra of the described functionalized supports.

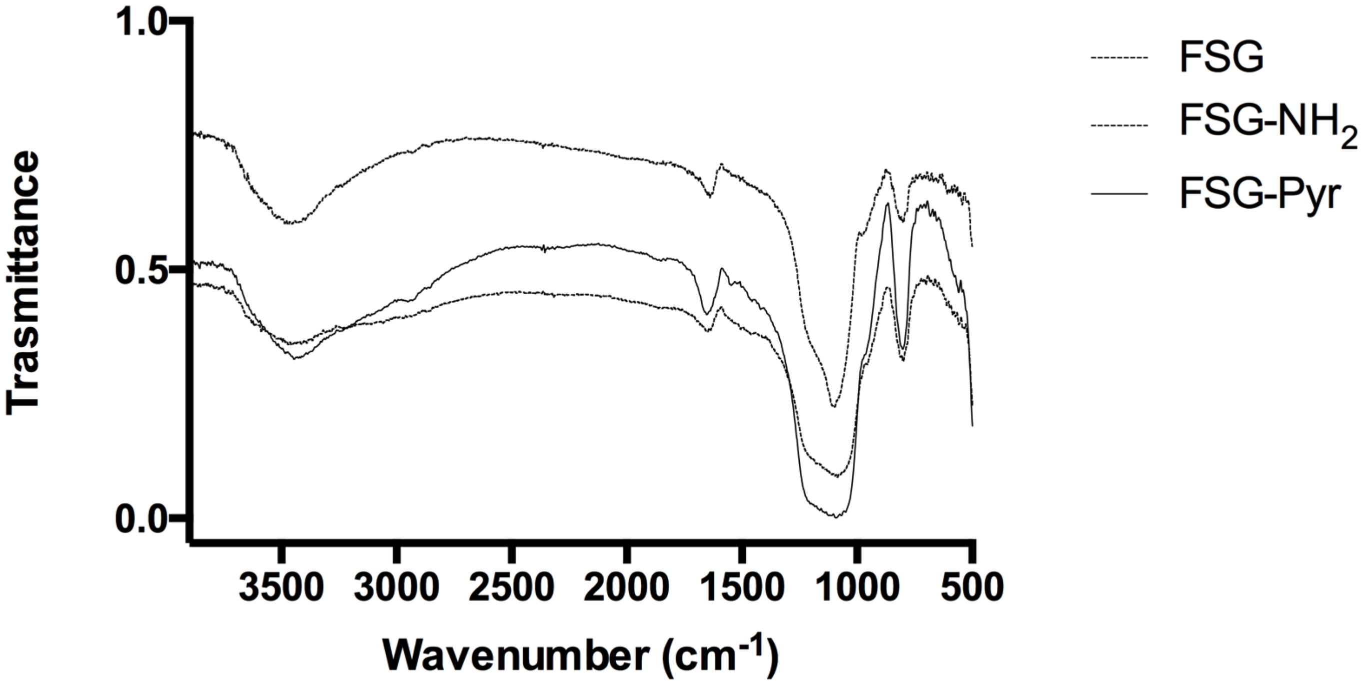

Figure S6. Catalytic activity in the presence of MPS as the oxidant, in the absence of any catalyst, at different pH values. Catalytic activity is expressed as % decolorization (i.e. % of initial absorbance at the λ_{max}).

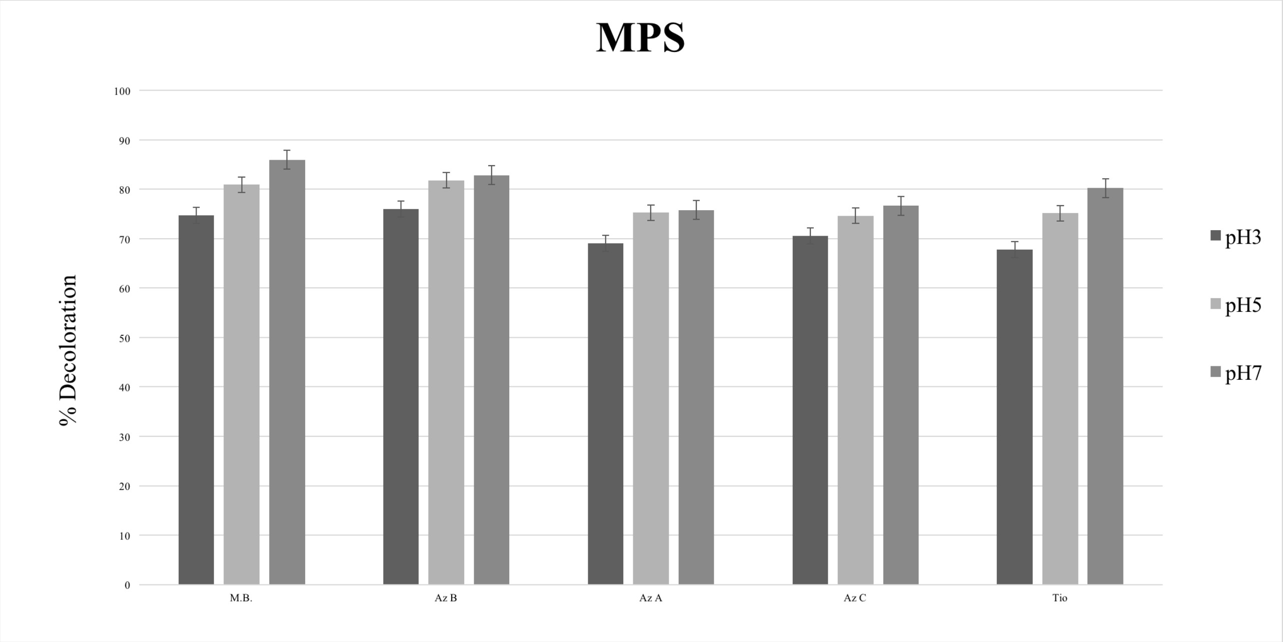

Supplement: Supplementary file 1 [file ijms-18-02553-s001.pdf]
